# Role of mu opioid receptor (MOR) agonist efficacy as a determinant of opioid antinociception in a novel assay of pain-depressed behavior in female and male mice

**DOI:** 10.3389/fpain.2023.1281698

**Published:** 2023-10-11

**Authors:** S. Stevens Negus, Hamid I. Akbarali, Minho Kang, Young K. Lee, Samuel A. Marsh, Edna J. Santos, Yan Zhang

**Affiliations:** ^1^ Department of Pharmacology & Toxicology, School of Medicine, Virginia Commonwealth University, Richmond, VA, United States; ^2^ Department of Medicinal Chemistry, School of Pharmacy, Virginia Commonwealth University, Richmond, VA, United States

**Keywords:** efficacy, analgesia, pain-depressed behavior, mice, locomotor, mu opioid agonist

## Abstract

**Introduction:**

Intermediate efficacy mu opioid receptor (MOR) agonists have potential to retain analgesic effectiveness while improving safety, but the optimal MOR efficacy for effective and safe opioid analgesia is unknown. Preclinical assays of pain-depressed behavior can assess effects of opioids and other candidate analgesics on pain-related behavioral depression, which is a common manifestation of clinically relevant pain and target of pain treatment. Accordingly, the present study goal was to validate a novel assay of pain-depressed locomotor behavior in mice and evaluate the role of MOR efficacy as a determinant of opioid analgesic effects and related safety measures.

**Methods:**

Male and female ICR mice were tested in a locomotor chamber consisting of 2 compartments connected by a doorway that contained a 1-inch-tall barrier. Dependent measures during 15-min behavioral sessions included crosses between compartments (which required vertical activity to surmount the barrier) and total movement counts (which required horizontal activity to break photobeams in each compartment).

**Results and Discussion:**

Intraperitoneal injection of lactic acid (IP acid) produced a concentration- and time-dependent depression of both endpoints. Optimal blockade of IP acid-induced behavioral depression with minimal motor impairment was achieved with intermediate-efficacy MOR treatments that also produced less gastrointestinal-transit inhibition and respiratory depression than the high-efficacy MOR agonist fentanyl. Sex differences in treatment effects were rare. Overall, these findings validate a novel procedure for evaluating opioids and other candidate analgesic effects on pain-related behavioral depression in mice and support continued research with intermediate-efficacy MOR agonists as a strategy to retain opioid analgesic effectiveness with improved safety.

## Introduction

Mu opioid receptor (MOR) ligands vary in their efficacy to activate MOR-coupled intracellular signaling pathways and downstream effects (1–4). High-efficacy MOR agonists such as fentanyl strongly activate MOR signaling and produce a full array of therapeutic effects (e.g., analgesia) and side effects (e.g., respiratory depression, sedation, constipation, abuse potential) (5). Lower efficacy MOR agonists can retain analgesic effects with fewer or less severe side effects; however, use of existing lower efficacy MOR agonists is complicated by factors that include metabolism to high-efficacy metabolites (e.g., buprenorphine) (6–8) or poor MOR selectivity leading to off-target side effects (e.g., kappa opioid receptor-mediated effects of nalbuphine) (9–14). These observations suggests that selective MOR agonists with MOR efficacy similar to or lower than that of buprenorphine might serve as viable candidate analgesics with improved safety relative to existing opioids.

Preclinical assessment of candidate analgesics has traditionally relied on procedures that measure “pain-stimulated” behaviors, which can be defined as behaviors that increase in rate, frequency, or intensity in the presence of a noxious stimulus (15). However, clinically relevant pain states often impair function and depress behavior, and clinical pain management often seeks to restore pain-depressed behaviors (16, 17). Accordingly, we and others have developed preclinical procedures to evaluate drug effects on “pain-depressed” behaviors, which can be defined as behaviors that decrease in rate, frequency, or intensity in the presence of a noxious stimulus (18–25). These procedures provide a translationally valid measure of pain-related behavioral depression and a relatively high degree of predictive validity in testing candidate analgesics (24, 26). MOR agonist analgesics produce antinociception in these procedures depending on variables that include noxious stimulus intensity and behavioral endpoint (23, 24, 27); however, the role of MOR efficacy has not been extensively examined (28).

Accordingly, the main goal of this study was to evaluate MOR efficacy as a determinant of opioid antinociception in a novel assay of pain-depressed behavior in male and female mice. Initial studies optimized experimental parameters and determined concentration-dependent effects of intraperitoneal injection of dilute lactic acid (IP acid) as a visceral noxious stimulus that mimics the tissue-acidification component of inflammatory pain (29, 30). Next, MOR efficacy was manipulated using two approaches. First, we tested single-molecule MOR agonists known to vary in MOR efficacy (fentanyl > morphine > buprenorphine > nalbuphine ≥ NAQ > naltrexone) (2, 31, 32). Second, we tested a series of fixed-proportion fentanyl/naltrexone mixtures that permit graded manipulation of net MOR efficacy and represented efficacy steps between buprenorphine and nalbuphine (32, 33).

As a complement to testing MOR-ligand antinociceptive effects in the assay of IP acid-induced behavioral depression, three additional studies were conducted. First, to assess sensitivity and selectivity of antinociception in this procedure to clinically effective analgesics, the effects of MOR ligands were compared to effects of a non-opioid positive-control analgesic (the non-steroidal anti-inflammatory drug ketoprofen) and a series of negative controls that are not approved clinically for acute pain treatment (diazepam, the kappa opioid receptor agonist U69593, psilocybin, and amphetamine). We predicted that only the clinically effective analgesics (MOR agonists, ketoprofen) would be effective to alleviate IP acid-induced behavioral depression. Second, to assess selectivity of MOR-agonist effectiveness to relieve behavioral depression induced by a pain stimulus, the effects of selected drugs were evaluated on behavioral depression produced by the nauseant agent lithium chloride (LiCl) as a non-pain stimulus (34, 35). We predicted that MOR agonists would fail to alleviate LiCl-induced behavior depression. Lastly, to compare the efficacy dependence of opioid antinociception with the efficacy dependence of common MOR-mediated side effects, fentanyl/naltrexone mixture effects were determined on measures of respiratory depression and gastrointestinal-transit inhibition (36). Overall, our results suggest that optimum relief of pain-depressed behavior with minimum side effects can be achieved with intermediate-efficacy MOR agonists and mixtures.

## Methods

### Subjects

Male and female ICR mice (Envigo, Frederick, MD) were 6–8 weeks old upon arrival to the laboratory, where they were single-housed in cages with corncob bedding (Envigo), a “nestlet” composed of pressed cotton (Ancare, Bellmore, NY), a cardboard tube for enrichment, and *ad libitum* access to water and food (Teklad LM-485 Mouse/Rat Diet; Envigo). Cages were mounted in racks in temperature-controlled rooms with a 12-h light/dark cycle in a facility approved by the American Association for Accreditation of Laboratory Animal Care. For mice in studies of locomotor activity and gastrointestinal transit, lights were on from 6:00 AM to 6:00 PM, and mice were tested during the light phase of the light/dark cycle. Mice in studies of respiration were housed on a reversed light/dark cycle (lights on from 6:00 PM to 6:00 AM), and tests were conducted during the dark phase of the cycle to reduce the probability of sleep and declining respiratory rates during testing as described previously (36, 37). All studies began at least 1 week after arrival at the laboratory and were usually completed during the second week after arrival. Animal-use protocols were approved by the Virginia Commonwealth University Institutional Animal Care and Use Committee and complied with the National Research Council Guide for the Care and Use of Laboratory Animals.

### Locomotor activity

#### Apparatus

**Figure 1:**
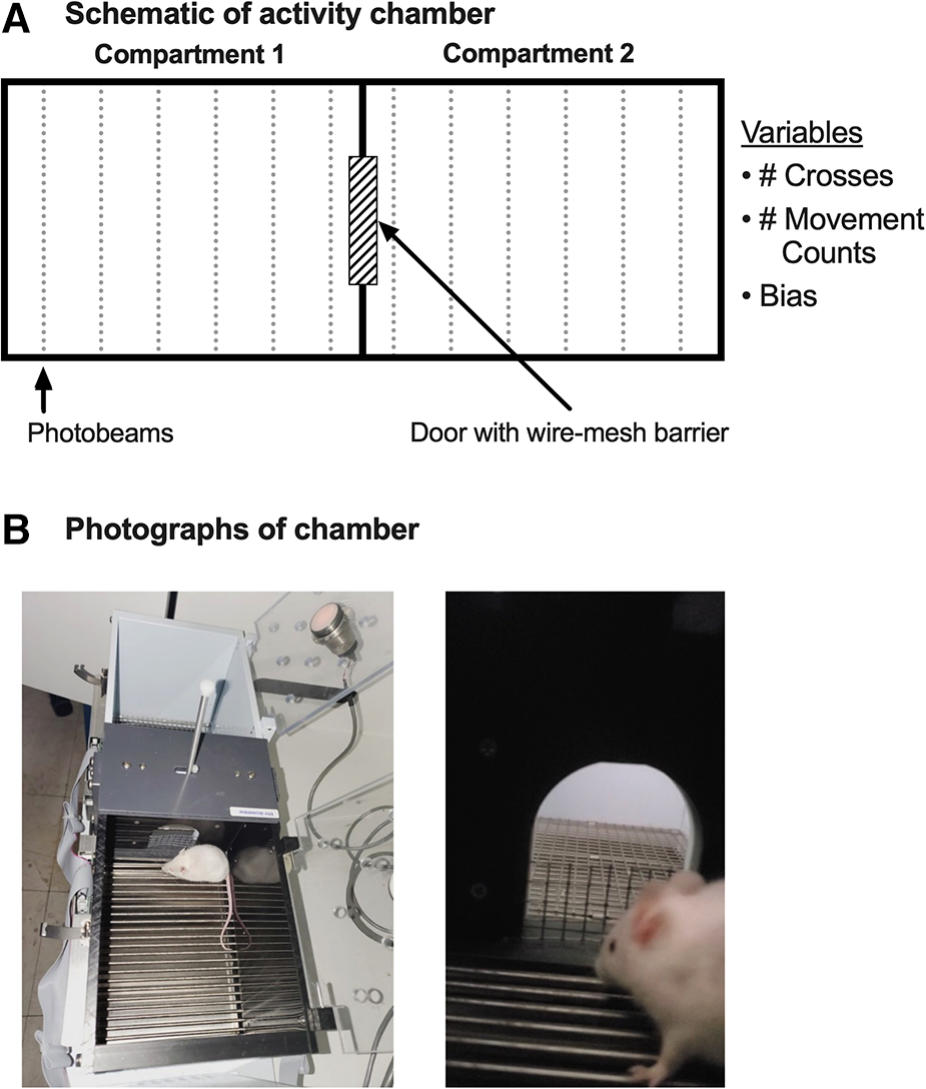
Schematic (**A**) and photographs (**B**) of the test chamber.

Horizontal locomotor activity was assessed in plexiglass and metal test boxes housed in sound-attenuating chambers (Med Associates, St. Albans, VT) and located in a procedure room separate from the housing room. Figure 1 shows that each box had two adjacent compartments (16.8 × 12.7 cm^2^ floor area × 12.7 cm high) separated by a wall. One compartment had black walls with a bar floor, and mice were always placed in this compartment to start each session. The other compartment had white walls with a wire-mesh floor. Additionally, each compartment had a clear plexiglass lid fitted with a house light as well as six photobeams arranged at 3-cm intervals across the long wall and 1 cm above the floor. Photobeams were monitored by a microprocessor operating Med Associates software. The wall separating the two compartments contained a central door (5 cm wide × 6 cm high). For most experiments, the lower 1-inch (2.54 cm) portion of the door was obstructed by a stainless-steel wire-mesh barrier that had to be surmounted for mice to cross back and forth between the two compartments.

#### Procedure

Initial parametric experiments were conducted to determine the session length, the height of the wire-mesh barrier in the doorway, and the potency and time course of IP acid to decrease behavior. For all subsequent studies with test drugs, the session length was 15 min, the barrier height was 1 in (2.54 cm), and a concentration of 0.56% IP acid was administered 5 min before experimental sessions. Each drug was tested under two conditions. First, vehicle and a range of drug doses was tested alone, with vehicle or drug being administered SC 30 min before the test session. Second, vehicle and a range of drug doses was tested as a pretreatment to IP acid. For these experiments, the test drug or its vehicle was administered SC 30 min before the session, and 0.56% lactic acid was administered IP 5 min before the session. The drugs and dose ranges were as follows: ketoprofen (0.01–10 mg/kg), fentanyl (0.01–0.32 mg/kg), morphine (1–32 mg/kg), buprenorphine (0.01–0.32 mg/kg), nalbuphine (1–32 mg/kg), NAQ (1–32 mg/kg), naltrexone (1.0 mg/kg), diazepam (0.1–10 mg/kg), U69593 (0.032–1 mg/kg), psilocybin (0.1–3.2 mg/kg), and amphetamine (0.32–10 mg/kg). Nalbuphine has been reported to produce some effects in mice mediated by kappa opioid receptors (12), and in an attempt to block these kappa effects and isolate MOR-mediated effects, an additional experiment evaluated effects of 10 mg/kg nalbuphine administered alone or as a pretreatment to IP acid 24 h after pretreatment with the long-acting kappa antagonist norbinaltorphimine (30 mg/kg SC).

As an additional strategy to manipulate the efficacy of MOR activation, a series of fixed-proportion fentanyl/naltrexone mixtures was also tested as we have described previously (32, 33). These fixed-proportion mixtures ranged from 56:1 fentanyl/naltrexone to 10:1 fentanyl/naltrexone, and each mixture was also tested across a range of doses (0.01–0.32 mg/kg fentanyl in combination with a corresponding naltrexone dose as determined by the fixed proportion). Each test drug or mixture was tested across a range from low doses that produced little or no change in behavior to high doses that (a) decreased behavior when the drug was tested alone, (b) significantly attenuated IP acid-induced depression of behavior, and/or (c) reached doses known to produce MOR antagonist effects in other studies (32).

To assess the selectivity of test drug effects on pain-related behavioral depression produced by IP acid, a subset of drugs was also evaluated for effects on behavioral depression produced by IP lithium chloride (IP LiCl) as a nauseant, non-pain stimulus (34, 35). An initial study evaluated effects of different IP LiCl doses (37.5–150 mg/kg) administered alone 5 min before test sessions. Subsequent experiments then evaluated effects of antinociceptive doses of morphine (3.2 mg/kg), buprenorphine (0.1 mg/kg), or amphetamine (3.2 mg/kg) administered SC 30 min before test sessions followed by IP 150 mg/kg LiCl 5 min before test sessions.

#### Dependent measures

Three dependent measures were determined for each session in each mouse: (1) “Crosses” defined as the number of crosses between the compartments and requiring mice to rear and surmount the vertical barrier in the doorway, (2) “Movement” defined as the total number of beam breaks summed across both compartments and requiring only horizontal locomotor activity, and (3) “Bias” defined as the proportion of each session spent on the side with black walls and the bar floor.

### Inhibition of gastrointestinal transit

#### Procedure

To evaluate the role of MOR efficacy as a determinant of opioid effects on gastrointestinal transit, the effects of fentanyl alone, naltrexone alone, and a series of fentanyl/naltrexone mixtures were determined as described previously using the charcoal-meal assay (36, 38). All mice were fasted for 24 h with free access to water during the entire fasting period and access to 5% dextrose for the first 8 h of the fasting period. Subsequently, separate groups of 12 ICR mice (6 male, 6 female) were used to test saline vehicle and each dose of fentanyl alone (0.01–0.32 mg/kg), naltrexone alone (0.32 mg/kg), and a series of fentanyl/naltrexone mixtures consisting of 0.32 mg/kg fentanyl + a naltrexone dose sufficient to produce proportions of 1:1 to 100:1 fentanyl/naltrexone (i.e., 0.32 mg/kg fentanyl + 0.32 mg/kg naltrexone for the 1:1 mixture and 0.32 mg/kg fentanyl + 0.0032 mg/kg naltrexone for the 100:1 mixture). In each group, mice received their designated treatment by SC injection, and 15 min later, they were given an oral gavage consisting of 5% aqueous suspension of charcoal (10 µl/g body weight; Sigma, C7606-125G) in a 10% gum Arabic solution. At 30 min after the administration of the charcoal meal, mice were euthanized by cervical dislocation, and the small intestine from the jejunum to the caecum was dissected and placed in cold saline to stop peristalsis.

#### Dependent measure

The fractional distance traveled by the leading edge of the charcoal meal in each mouse was normalized to the total length of the small intestine in that mouse using the equation Fractional Distance = (Charcoal Distance)/(Small Intestinal Length). Data from each mouse were then expressed as a percentage of the mean Fractional Distance in the saline control group using the equation % Saline Value = [(Fractional Distance after Treatment in a Given Mouse)/(Mean Fractional Distance in Saline Group)] × 100.

### Respiratory depression

#### Procedure

To evaluate the role of MOR efficacy as a determinant of opioid effects on breathing, the effects of fentanyl alone, naltrexone alone, and a series of fentanyl/naltrexone mixtures were determined as described previously using whole-body plethysmography chambers (Data Sciences International, St. Paul, MN) to measure respiratory rate (breath frequency), breath tidal volume, and minute volume (36, 37, 39). Respiratory studies were conducted under 660 nm illumination, a wavelength with limited visibility to mice, to promote maintenance of subjects in the dark phase of their cycle. As noted above under “Subjects”, testing mice during their active phase (dark cycle) promoted higher basal respiration rates and prevented mice from sleeping during the procedure. Mice were placed in the chambers for a 30-min habituation session the day before testing under ambient air conditions. On the test day, plethysmography chambers were supplied with 5% CO_2_, 21% O_2_, and a balance of N_2_. This CO_2_-enriched mixture has been found to minimize ventilatory variability over time, improve sensitivity for detecting opioid-induced respiration depression, and is devoid of anxiogenic effects (37, 40, 41). Separate groups of 12 ICR mice (6 male, 6 female) were used to test saline vehicle and each dose of fentanyl alone (0.01–1.0 mg/kg), naltrexone alone (0.32 mg/kg), and a series of fentanyl/naltrexone mixtures consisting of 0.32 mg/kg fentanyl + a naltrexone dose sufficient to produce proportions of 1:1–100:1 fentanyl/naltrexone. In each group, mice received their designated treatment by SC injection before being placed into the plethysmography chamber for a 30-min session.

#### Dependent measures

The primary dependent measures recorded were breath frequency per minute, tidal volume in ml per breath, and minute volume in ml/min (i.e., breath frequency per minute × tidal volume) during 1 min bins of the 30-min session. Previous studies (37) and preliminary data indicated that fentanyl effects peaked within 10 min and lasted for at least 30 min. Accordingly, the primary dependent measure for data analysis was mean minute volume in each mouse from 10 to 30 min after injection (MV_10–30′_). Data from each mouse were then expressed as a percentage of the mean MV_10–30′_ in the saline control group using the equation % Saline Value = [(MV_10–30′_ after Treatment in a Given Mouse)/(Mean MV_10–30′_ in Saline Group)] × 100.

### Data analysis

For each endpoint of each procedure, data were averaged across mice within a given treatment and submitted to analysis that proceeded in three steps as we have described previously for preclinical studies that include both sexes but are not intended to examine sex as the primary variable of interest (32, 42). First, data for a given manipulation were pooled across sexes and analyzed by one-way ANOVA. A significant ANOVA was followed by either Dunnett's *post hoc* test to compare test treatments with vehicle treatment or Tukey's *post hoc* test to compare all treatments with each other. Second, data were segregated by sex and analyzed by two-way ANOVA, with sex as one of the variables. A significant main effect of Sex or Sex × Treatment interaction was followed by a Holm-Sidak *post hoc* test. Lastly, two-way ANOVA results were submitted to *post hoc* power analyses to calculate the Cohen's f effect size, achieved power (1 - β), and the total number of animals predicted as necessary to achieve power ≥0.8. This *post hoc* power analysis was included to provide guidance for future studies that might investigate sex as a primary variable of interest. Prism 9.0 (GraphPad) was used for all ANOVAs, and the criterion for significance was *p* < 0.05. G*power (43) was used for all *post hoc* power analyses.

### Drugs

Fentanyl HCl, morphine sulfate, buprenorphine HCl, naltrexone HCl, U69593, psilocybin, and amphetamine sulfate were provided by the National Institute on Drug Abuse Drug Supply Program and were dissolved in sterile saline. Nalbuphine HCl (provided by Dr. Kenner Rice, National Institute on Drug Abuse Intramural Research Program) was also dissolved in sterile saline. NAQ {17-Cyclopropyl-methyl-3,14β-dihydroxy-4,5α-epoxy-6α-[(3′-isoquinolyl)acetamido]-morphinan; provided by Dr. Yan Zhang, Virginia Commonwealth University} was dissolved in 10% DMSO and 90% water. Ketoprofen (100 mg/ml; Ford Dodge, IA) was diluted in sterile saline. Diazepam (5 mg/ml, Hospira, Lake Forest, IL) was diluted in 1:4:5 ethanol, propylene glycol, and saline. All drugs were administered subcutaneously (SC) in volumes of 10 ml/kg. Lactic acid and lithium chloride were purchased from Sigma-Aldrich (St. Louis, MO). Lactic acid was diluted in sterile water, while lithium chloride was dissolved in saline. Both were administered intraperitoneally (IP) in a volume of 10 ml/kg.

## Results

### Locomotor effects

Figure 1 shows a diagram of the 2-compartment experimental chamber used to evaluate three dependent measures of locomotor activity: (1) the number of “Crosses” between compartments, (2) the number of “Movement Counts” (photobeam breaks) summed across both compartments, and (3) “Bias” between compartments expressed as the fraction of total session time spent in the compartment with black walls and a bar floor. Initial parametric studies summarized in Supplementary Figure S1 were used to determine the session length (15 min) and barrier height between the chambers (1 in) that were used for all other studies.

**Figure 2:**
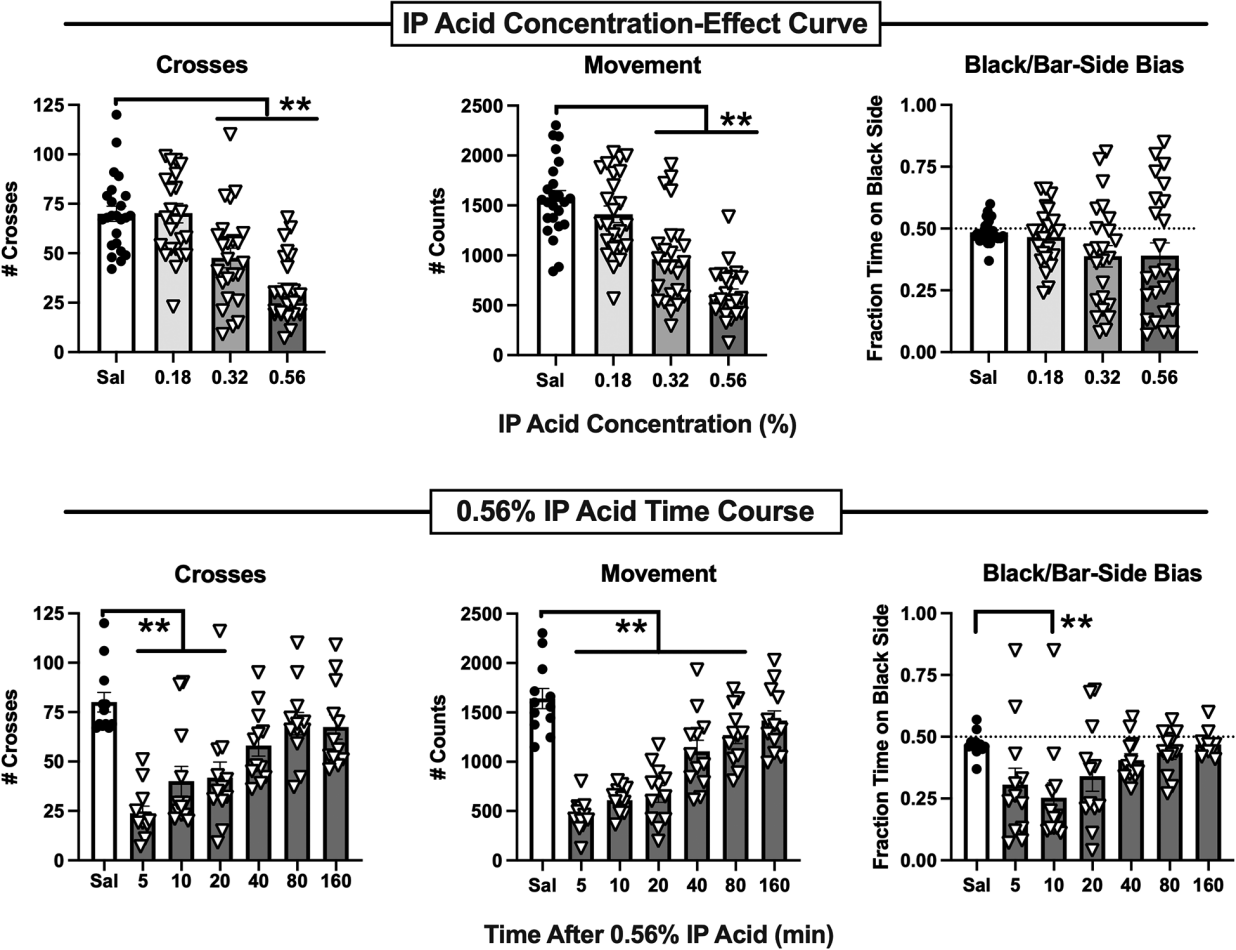
Potency and time course of IP acid-induced behavioral depression. Top panels show the IP acid concentration-effect curve for Crosses (left), Movement Counts (center), and Bias (right). IP acid was administered 5 min before 15-min test sessions. Bottom panels show the time course of effects produced by 0.56% IP acid on Crosses (left), Movement Counts (center), and Bias (right). IP acid was administered at the designated intervals before 15-min test sessions. Each bar shows the mean ± SEM for 12 mice (6 male, 6 female), and points show data for individual mice. **p < 0.01 for comparison between the designated groups.

Figure 2 shows that intraperitoneal administration of dilute lactic acid (IP acid) administered 5 min before the session produced a concentration-dependent decrease in both crosses [F(3,92) = 18.94, *p* < 0.0001] and movement [F(3,92) = 31.71, *p* < 0.0001], while bias was not significantly affected. In *post hoc* testing, both 0.32 and 0.56% IP acid significantly decreased both crosses and movement. Figure 2 also shows a time-dependent effect of 0.56% IP acid administered at different pretreatment intervals before the session on crosses [F(6,77) = 10.78, *p* < 0.0001], movement [F(6,77) = 28.22, *p* < 0.0001], and bias [F(6,77) = 3.631, *p* = 0.0032]. Post hoc analysis indicated a decrease in crosses at pretreatment times of 5–20 min, movement counts at pretreatment times of 5–80 min, and bias only at a pretreatment time of 10 min. When sex was included as a variable in two-way ANOVA for data in each panel of Figure 2, there was not a significant Sex × Concentration or Sex × Time interaction on any endpoint. However, there was a significant main effect of Sex for IP acid concentration [F(1,88) = 8.40, *p* < 0.0047] and pretreatment time [F(1,70) = 17.93, *p* < 0.0001] on crosses, with males showing a higher number of crosses than females. Taken together, these findings indicated that IP acid produced a pain-related depression of crosses and movement, and subsequent studies evaluated effectiveness of drugs to alleviate pain-related behavioral depression produced by 5 min pretreatment with 0.56% IP acid.

A total of 15 different drugs or drug mixtures were evaluated for their effects when administered both alone and as a pretreatment to 0.56% IP acid, and testing with each drug included control treatments with drug vehicle alone and drug vehicle + 0.56% IP acid. There were no significant differences in crosses, movement counts, or bias scores for the vehicle-alone controls across all 15 drugs, indicating that these behavioral measures remained relatively stable across multiple cohorts of mice tested over more than a year. Additionally, Table 1 shows that, relative to the vehicle-alone treatment, vehicle + 0.56% IP acid produced a significant decrease in both crosses and movement for each drug. As in the initial evaluation of IP acid potency and time course, bias was usually not significantly affected, but significant decreases in bias (i.e., reduced preference for the black-wall/bar-floor compartment) were apparent in some groups. Because bias was not reliably altered by 0.56% IP acid treatment, the analysis of drug effects focused only on IP acid-induced depression of crosses and movement. Control data for vehicle alone and vehicle + 0.56% IP acid were also collapsed across all 15 drugs and submitted to two-way ANOVA with IP acid treatment and sex as the two factors. Results are shown in Supplementary Figure S2. With all 180 mice included in the analysis (90 male, 90 female), IP acid significantly decreased crosses [F(1,356) = 318, *p* < 0.0001], movement [F(1,356) = 1071, *p* < 0.0001], and bias [F(1,356) = 30.56, *p* < 0.0001]. Additionally, there was a main effect of sex on crosses [F(1,356) = 25.77, *p* < 0.0001] and bias [F(1,356) = 5.768, *p* = 0.0168], with males showing slightly more crosses and slightly higher bias scores than females. However, there was not a significant Sex × IP acid interaction on any endpoint, further supporting an absence of sex differences in IP acid effects.

**Table 1:** *P*-values for *t*-test comparisons of effects produced by vehicle alone vs. vehicle + 0.56% IP acid in cohorts of mice used for each of the 15 drugs evaluated in this study.

Drug cohort	Crosses	Movement	Bias
Ketoprofen	0.0045*	<0.0001*	0.1837
Fentanyl	0.0001*	<0.0001*	0.9408
Morphine	0.0224*	<0.0001*	0.0009*
Buprenorphine	0.0004*	<0.0001*	0.0891
Nalbuphine	<0.0001*	<0.0001*	0.0716
NAQ	0.0007*	<0.0001*	0.1139
Naltrexone	<0.0001*	<0.0001*	0.1348
56:1 Fent/NTX	<0.0001*	<0.0001*	0.3097
32:1 Fent/NTX	<0.0001*	<0.0001*	0.8595
18:1 Fent/NTX	<0.0001*	<0.0001*	0.4475
10:1 Fent/NTX	0.0007*	<0.0001*	<0.0001*
Diazepam	0.0113*	<0.0001*	0.0161*
U69593	0.0003*	<0.0001*	0.0001*
Psilocybin	<0.0001*	<0.0001*	0.0354*
Amphetamine	<0.0001*	<0.0001*	0.0789

Relative to effects of vehicle alone, vehicle + 0.56% IP acid significantly decreased crosses and movement in each cohort of mice, whereas bias was significantly affected in only a subset of cohorts.

* Asterisk indicates that *p*-value met the criterion for significance of *p* < 0.05.

**Figure 3:**
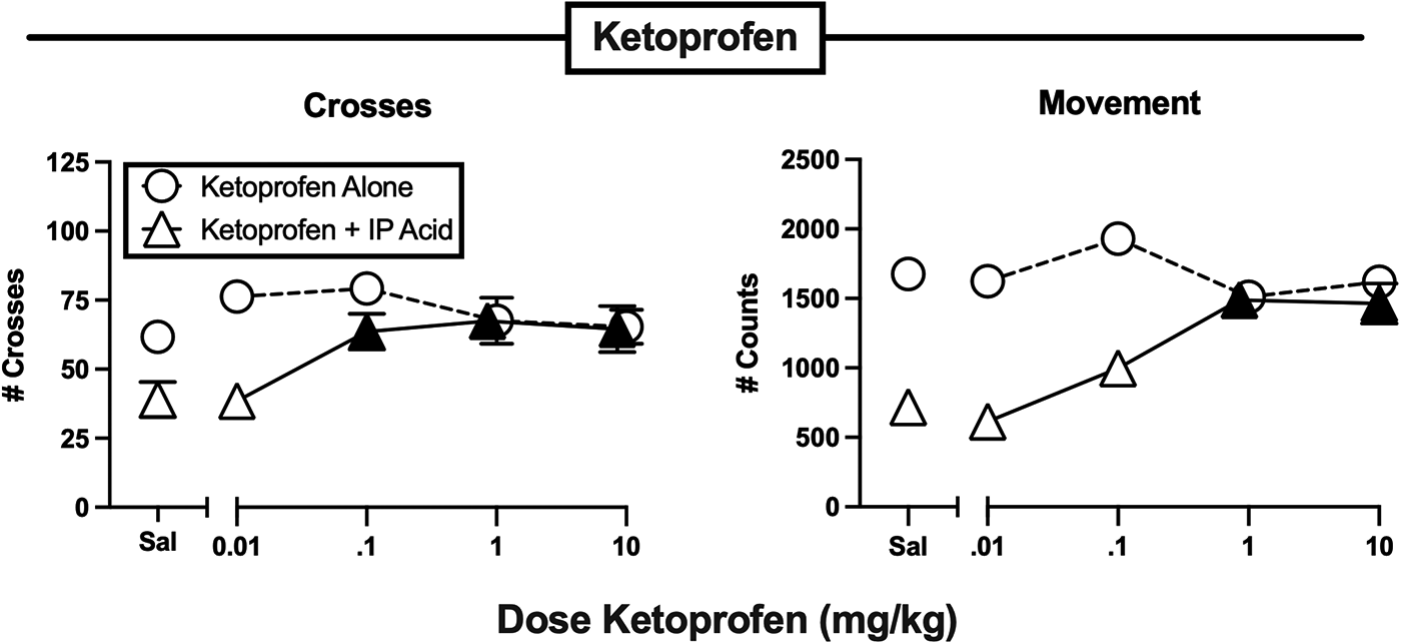
Effects of the NSAID ketoprofen administered alone or as a pretreatment to 0.56% IP acid. Abscissae: Dose ketoprofen in mg/kg SC administered 30 min before test sessions. If IP acid was administered, it was injected 5 min before test sessions. Ordinates: Number of Crosses (left panel) or Movement Counts (right panel) during the 15-min session. Each point shows mean ± SEM for 12 mice (6 male, 6 female). Filled points indicate significantly different from “Sal” as determined by one-way ANOVA followed by Dunnett's *post hoc* test, *p* < 0.05. Filled triangles indicate an antinociceptive effect of ketoprofen.

**Figure 4:**
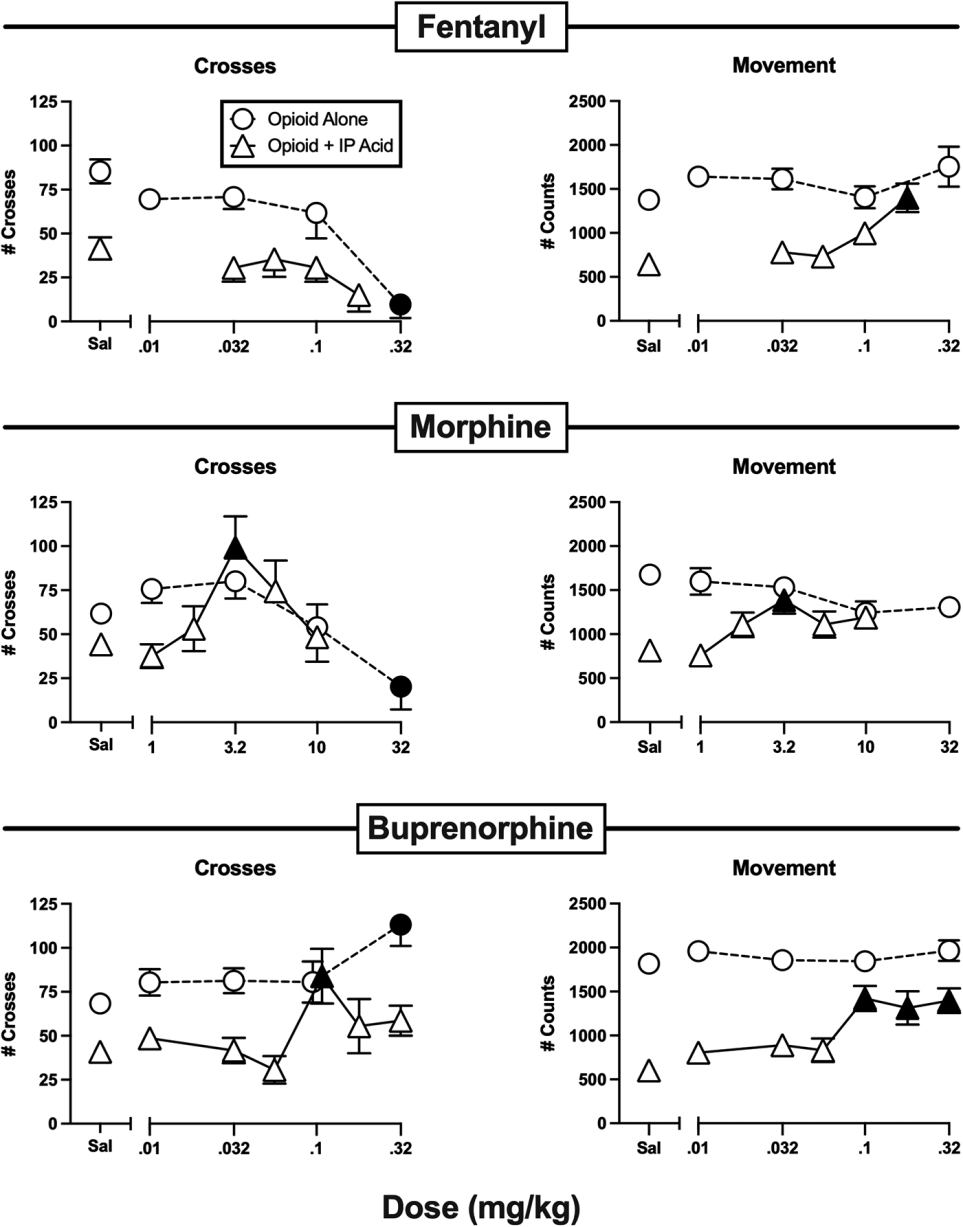
Effects of the opioids fentanyl, morphine, and buprenorphine administered alone or as a pretreatment to 0.56% IP acid. Abscissae: Dose in mg/kg SC administered 30 min before test sessions. If IP acid was administered, it was injected 5 min before test sessions. Ordinates: Number of Crosses (left panels) or Movement Counts (right panels) during the 15-min session. Each point shows mean ± SEM for 12 mice (6 male, 6 female). Filled points indicate significantly different from “Sal” as determined by one-way ANOVA followed by Dunnett's *post hoc* test, *p* < 0.05. Filled triangles indicate an antinociceptive effect.

**Figure 5:**
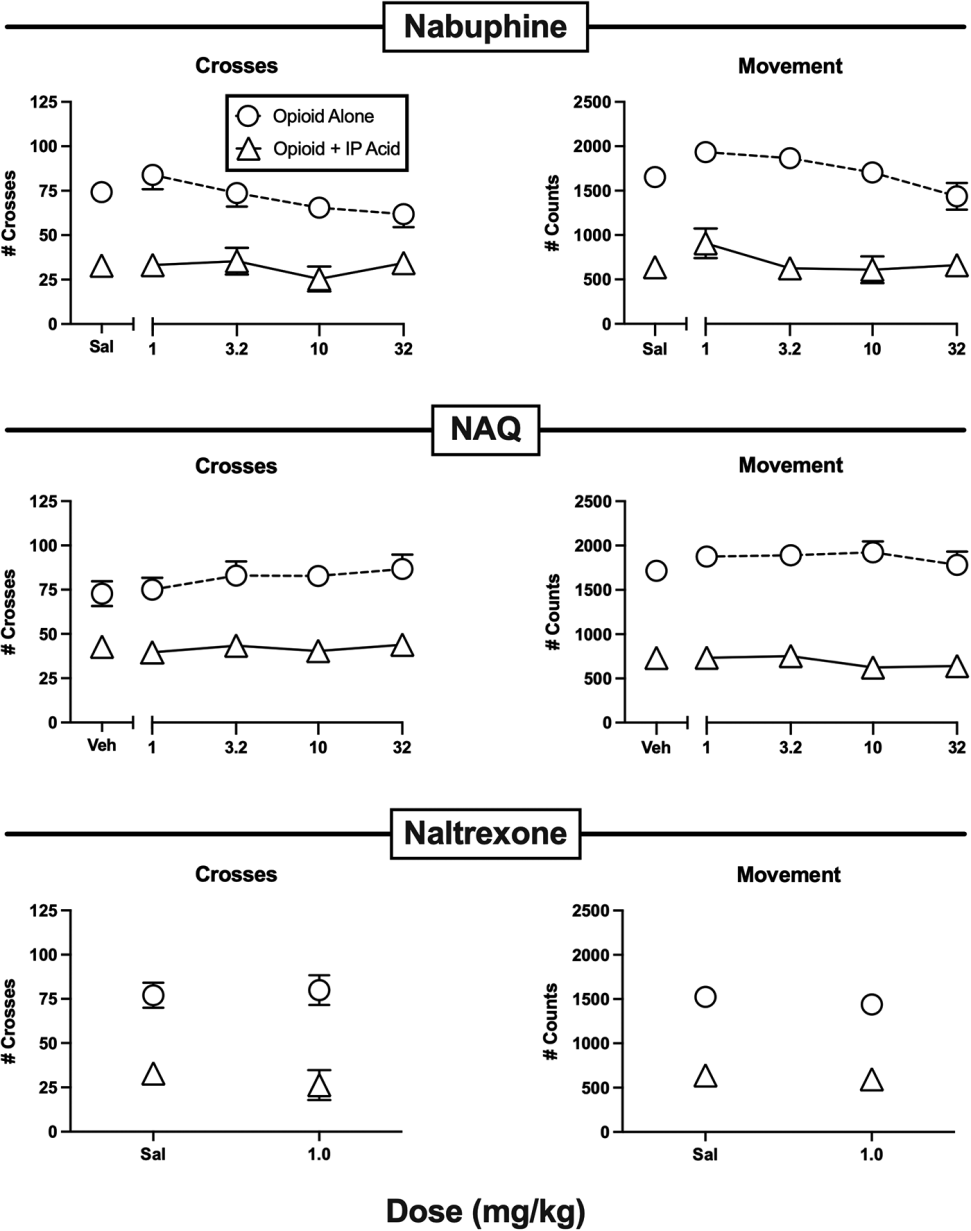
Effects of the opioids nalbuphine, NAQ, and naltrexone administered alone or as a pretreatment to 0.56% IP acid. Abscissae: Dose in mg/kg SC administered 30 min before test sessions. If IP acid was administered, it was injected 5 min before test sessions. Ordinates: Number of Crosses (left panels) or Movement Counts (right panels) during the 15-min session. Each point shows mean ± SEM for 12 mice (6 male, 6 female).

**Figure 6:**
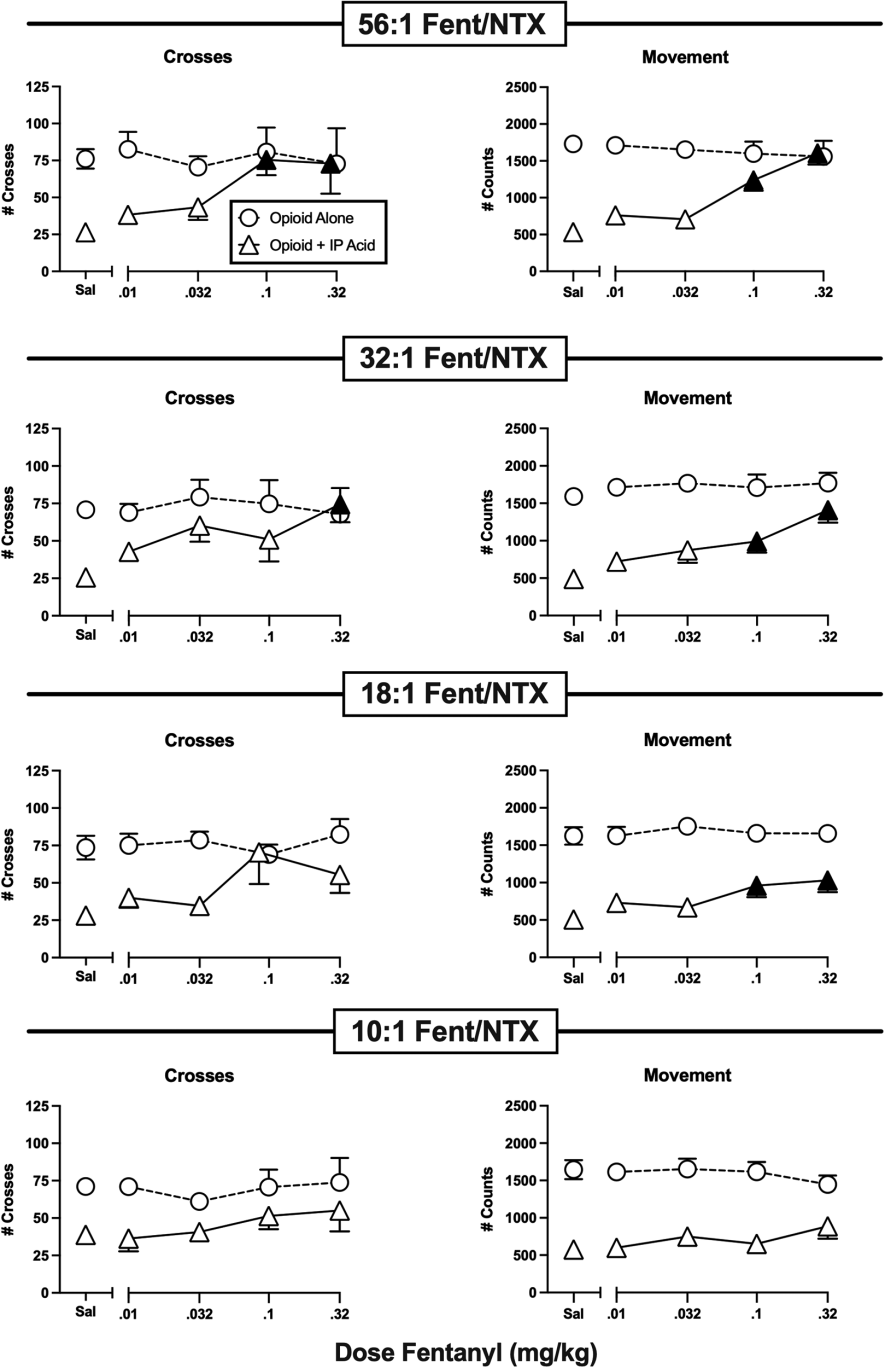
Effects of fentanyl/naltrexone mixtures administered alone or as a pretreatment to 0.56% IP acid. Abscissae: Dose in mg/kg SC administered 30 min before test sessions. If IP acid was administered, it was injected 5 min before test sessions. Ordinates: Number of Crosses (left panels) or Movement Counts (right panels) during the 15-min session. Each point shows mean ± SEM for 12 mice (6 male, 6 female). Filled points indicate significantly different from “Sal” as determined by one-way ANOVA followed by Dunnett's *post hoc* test, *p* < 0.05. Filled triangles indicate an antinociceptive effect.

**Figure 7:**
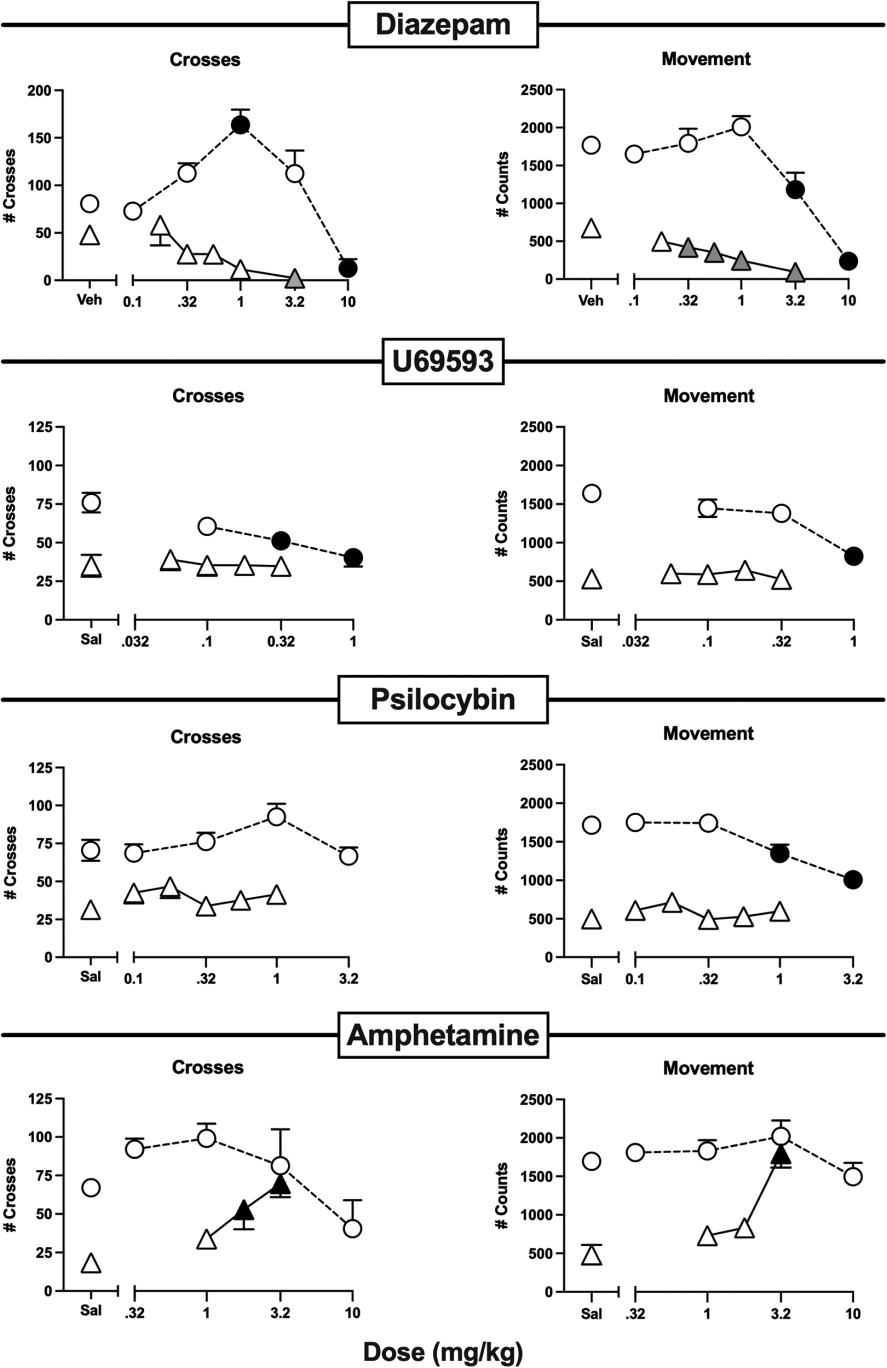
Effects of the non-analgesic negative controls diazepam, U69593, psilocybin, and amphetamine administered alone or as a pretreatment to 0.56% IP acid. Abscissae: Dose in mg/kg SC administered 30 min before test sessions. If IP acid was administered, it was injected 5 min before test sessions. Ordinates: Number of Crosses (left panels) or Movement Counts (right panels) during the 15-min session. Each point shows mean ± SEM for 12 mice (6 male, 6 female). Filled points indicate significantly different from “Sal” as determined by one-way ANOVA followed by Dunnett's *post hoc* test, *p* < 0.05. Filled gray triangles in “Diazepam” indicate a significant exacerbation of IP acid-induced depression. Filled black triangles in “Amphetamine” indicate an antinociceptive effect.

Figures 3–7 show effects of drugs and drug mixtures administered alone or as a pretreatment to 0.56% IP acid on crosses and movement. Table 2 shows one-way ANOVA or t-test results for effects of each drug administered alone or as a pretreatment to 0.56% IP acid on both crosses and movement. The nonsteroidal anti-inflammatory drug ketoprofen was tested as a non-opioid positive control, and Figure 3 shows that ketoprofen had no effect on either crosses or movement when it was administered alone, but it dose-dependently alleviated IP acid-induced depression of both crosses and movement. In this and all other figures, a black-filled triangle indicates an antinociceptive effect of the test drug to relieve IP acid-induced behavioral depression.

**Table 2:** One-way ANOVA or t-test results for each drug administered alone or as a pretreatment to 0.56% IP acid on crosses and movement.

Drug	± IP acid	Crosses	Movement	Figure
Ketoprofen	Alone	F(4,55) = 1.65, *p* = 0.1742	F(4,55) = 4.33, *p* = 0.0041^^^	3
	+ IP acid	F(4,55) = 5.01, *p* = 0.0016*	F(4,55) = 18.02, *p* < 0.0001*-F	
Fentanyl	Alone	F(4,55) = 11.12, *p* < 0.0001*	F(4,55) = 1.28, *p* = 0.2874	4
	+ IP acid	F(4,55) = 1.36, *p* = 0.2599	F(4,55) = 7.79, *p* < 0.0001*	
Morphine	Alone	F(4,55) = 4.86, *p* = 0.0020*	F(4,55) = 2.28, *p* = 0.0726	4
	+ IP acid	F(5,66) = 3.02, *p* = 0.0162*	F(5,66) = 3.27, *p* = 0.0107*	
Buprenorphine	Alone	F(4,55) = 3.44, *p* = 0.0139*	F(4,55) = 0.48, *p* = 0.7481	4
	+ IP acid	F(6,77) = 2.92, *p* = 0.0126*	F(6,77) = 6.83, *p* < 0.0001*	
Nalbuphine	Alone	F(4,55) = 1.78, *p* = 0.1453	F(4,55) = 3.25, *p* = 0.0184^^^	5
	+ IP acid	F(4,55) = 0.47, *p* = 0.7610	F(4,55) = 0.95, *p* = 0.4424	
NAQ	Alone	F(4,55) = 0.72, *p* = 0.5850	F(4,55) = 0.66, *p* = 0.6208	5
	+ IP acid	F(4,55) = 0.12, *p* = 0.9742-m	F(4,55) = 0.87, *p* = 0.4902-F	
Naltrexone	Alone	*t *= 0.27, *df *= 22, *p* = 0.7934-m	*t *= 0.64, *df *= 22, *p* = 0.5305	5
	+ IP acid	*t *= 0.67, *df *= 22, *p* = 0.5079	*t *= 0.26, *df *= 22, *p* = 0.7952	
56:1 Fent/NTX	Alone	F(4,55) = 0.12, *p* = 0.9742	F(4,55) = 0.27, *p* = 0.8972	6
	+ IP acid	F(4,55) = 3.729, *p* = 0.0094*	F(4,55) = 14.59, *p* < 0.0001*-m	
32:1 Fent/NTX	Alone	F(4,55) = 0.14, *p* = 0.9648	F(4,55) = 0.35, *p* = 0.8408	6
	+ IP acid	F(4,55) = 3.07, *p* = 0.0236*	F(4,55) = 6.51, *p* = 0.0002*	
18:1 Fent/NTX	Alone	F(4,55) = 0.42, *p* = 0.7968	F(4,55) = 0.31, *p* = 0.8704	6
	+ IP acid	F(4,55) = 2.17, *p* = 0.0841	F(4,55) = 3.44, *p* = 0.0139*	
10:1 Fent/NTX	Alone	F(4,55) = 0.25, *p* = 0.9080	F(4,55) = 0.45, *p* = 0.7745-F	6
	+ IP acid	F(4,55) = 0.81, *p* = 0.5222	F(4,55) = 1.45, *p* = 0.2290	
Diazepam	Alone	F(5,66) = 12.98, *p* < 0.0001*	F(5,66) = 19.24, *p* < 0.0001*-f	7
	+ IP acid	F(5,66) = 4.20, *p* = 0.0022*	F(5,66) = 10.55, *p* < 0.0001*	
U69593	Alone	F(3,44) = 7.54, *p* = 0.0004*-M	F(3,44) = 13.38, *p* < 0.0001*-f	7
	+ IP acid	F(4,55) = 0.08, *p* = 0.9887	F(4,55) = 0.40, *p* = 0.8099	
Psilocybin	Alone	F(4,55) = 2.45 *p* = 0.0569	F(4,55) = 11.92, *p* < 0.0001*-m	7
	+ IP acid	F(5,66) = 1.16, *p* = 0.3359	F(5,66) = 1.80, *p* = 0.1241	
Amphetamine	Alone	F(4,55) = 2.57, *p* = 0.0478^^^	F(4,55) = 1.94, *p* = 0.1368	7
	+ IP acid	F(3,44) = 6.88, *p* = 0.0007*	F(3,44) = 19.10, *p* < 0.0001*	

Upper case letters (M,F) or lower case letters (m,f) indicate a Sex × Dose interaction (upper case) or main effect of Sex (lower case), with the letter indicating whether males (M,m) or females (F,f) showed higher scores. See Supplementary Data for detailed report of statistical analysis for sex differences.

* Asterisk indicates a significant drug effect with Dunnett's *post hoc* test showing that at least one drug dose produced an effect different from vehicle as indicated by filled points in Figures 3–7.

^ Caret indicates a significant ANOVA but no difference between vehicle and drug doses by Dunnett's *post hoc* test.

Figure 4 shows the effects of the higher MOR efficacy opioid analgesics fentanyl, morphine, and buprenorphine. Fentanyl alone dose-dependently decreased crosses while having no effect on movement at the doses tested. At doses below those that decreased crosses, fentanyl failed to alleviate IP acid-induced depression of crosses, but it did alleviate IP acid-induced depression of movement at a high dose of 0.18 mg/kg. Like fentanyl, morphine alone also decreased crosses without affecting movement. However, when morphine was tested at doses below those that decreased crosses, it significantly alleviated IP acid-induced depression of both crosses and movement with an inverted-U shaped dose-effect curve that peaked at 3.2 mg/kg. Buprenorphine alone significantly increased crosses at the highest dose tested, but it did not alter movement. Like morphine, buprenorphine also significantly alleviated IP acid-induced depression of both crosses (at 0.1 mg/kg) and movement (at 0.1–0.32 mg/kg).

Figure 5 shows the effects of the lower MOR efficacy opioids nalbuphine, NAQ, and naltrexone. None of these compounds significantly altered crosses or movement when administered alone, and they also did not significantly alleviate IP acid-induced depression of crosses or movement. A dose of 10 mg/kg nalbuphine was also tested 24 hr after pretreatment with 32 mg/kg norbinaltorphimine to block kappa opioid receptors, but this treatment did not alter nalbuphine effects, suggesting that kappa receptor-mediated effects did not mask MOR-mediated antinociception (data not shown).

Figure 6 shows the effects of graded fixed-proportion mixtures of fentanyl and naltrexone. We have reported previously that declining proportions of fentanyl to naltrexone in these mixtures can be used to produce declining levels of net MOR efficacy (32, 33, 44–46). In this case, the range of mixtures from 56:1 to 10:1 fentanyl/naltrexone represents a range of MOR efficacies approximately equal to the range between buprenorphine and nalbuphine as single-molecule opioids (32, 33). When administered alone, none of these mixtures significantly altered either crosses or movement; however, they displayed graded effectiveness to alleviate IP acid-induced depression of crosses and movement. Thus, the mixture with the highest fentanyl proportion (56:1), and hence the highest MOR efficacy, fully blocked IP acid-induced depression of both crosses and movement. The 32:1 mixture produced similar effects, whereas the 18:1 mixture significantly alleviated only IP acid-induced depression of movement, and the lowest 10:1 mixture was not effective to alleviate IP acid-induced depression of either crosses or movement. Taken together with effects of the single-molecule opioids shown in Figures 4, 5, these results suggest that optimum relief of pain-depressed behavior in this assay was accomplished with intermediate-efficacy MOR agonists or mixtures with efficacy low enough to avoid severe motor impairment but high enough to produce antinociception.

Figure 7 shows the effects of a series of drugs that are not approved as analgesics. Diazepam, U69593, and psilocybin all decreased crosses and/or movement when high doses were administered alone, and an intermediate 1.0 mg/kg dose of diazepam alone also produced a striking increase in crosses. However, when tested at doses below those that decreased behavior when administered alone, none of these compounds alleviated IP acid-induced depression of crosses or movement. Indeed, diazepam only exacerbated IP acid-induced depression of both crosses and movement, even when tested at the 1.0 mg/kg dose that robustly increased crosses when it was administered alone. In contrast to results with these compounds, amphetamine did not significantly alter crosses or movement when it was administered alone, but it dose-dependently alleviated IP acid-induced depression of both crosses and movement.

Sex differences in drug effects were rare and were not systematically related to drug mechanism of action, MOR efficacy, or presence/absence of IP acid. Table 2 shows that, of the 60 different dose-effect curves in Figures 3–7 (15 drugs × 2 endpoints/drug × absence/presence of IP acid), there was a significant Sex × Dose interaction in four cases, and a significant main effect of Sex in six cases. When sex differences were observed, males exhibited more crosses, and females usually exhibited more movement counts. Supplementary Tables S1–S15 show detailed two-way ANOVA results and *post hoc* power analyses to address sex as a biological variable for each dose-effect curve in Figures 3–7.

**Figure 8:**
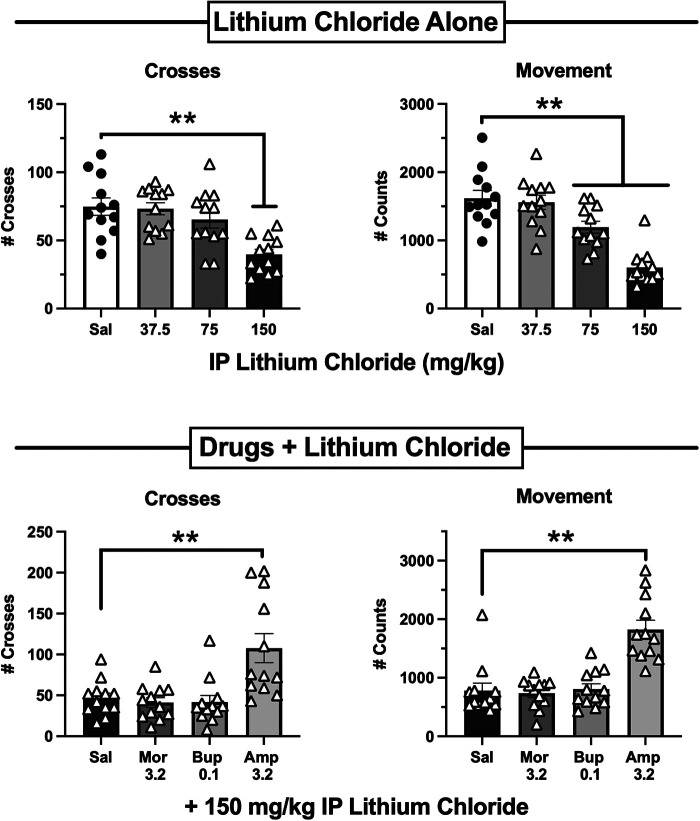
Effects of lithium chloride (liCl) administered alone or after pretreatment with 3.2 mg/kg morphine, 0.1 mg/kg buprenorphine, or 3.2 mg/kg amphetamine. Top panels show the LiCl dose-effect curve for Crosses (left) and Movement Counts (right). LiCl was administered IP 5 min before 15-min test sessions. Bottom panels show Crosses (left) and Movement Counts (right) after administration of saline or test drugs as a pretreatment to 150 mg/kg LiCl. Each bar shows the mean ± SEM for 12 mice (6 male, 6 female), and points show data for individual mice. **p < 0.01 for comparison between the designated groups.

Both MOR agonists and amphetamine can stimulate locomotor activity in mice under some conditions [e.g., (32, 47)], raising the possibility that their alleviation of IP acid-induced depression of crosses or movement might have reflected non-selective locomotor stimulation rather than analgesia. To address this issue, antinociceptive doses of morphine (3.2 mg/kg), buprenorphine (0.1 mg/kg), and amphetamine (3.2 mg/kg) were evaluated for their effects on behavior depressed by lithium chloride (LiCl) as a non-pain stimulus. Figure 8 (top panels) show that, as with IP acid, IP administration of 37.5–150 mg/kg LiCl 5 min before behavioral sessions produced a dose-dependent decrease in both crosses [F(3,44) = 9.35, *p* < 0.0001] and movement [F(3,44) = 23.09, *p* < 0.0001]. However, Figure 8 (bottom panels) shows that behavioral depression of both crosses [F(3,44) = 9.16, *p* < 0.0001] and movement [F(3,44) = 20.04, *p* < 0.0001] elicited by 150 mg/kg LiCl was blocked only by amphetamine but not by either morphine or buprenorphine. These results suggest that morphine and buprenorphine blockade of IP acid-induced behavioral depression cannot be attributed to non-selective behavioral stimulation. Supplementary Tables S16, S17 show that there were no significant sex differences in effects of LiCl or test drugs + LiCl.

**Figure 9:**
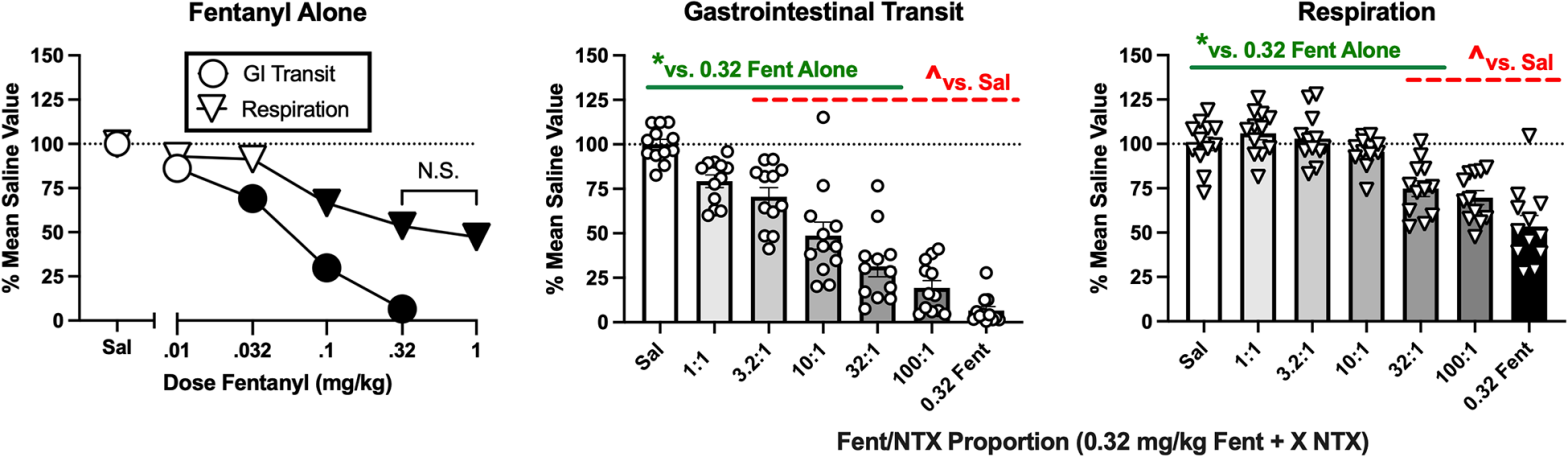
Effects of fentanyl alone or of fentanyl/naltrexone mixtures on gastrointestinal transit and respiration. Left panel shows effects of saline (Sal) or increasing doses of fentanyl alone. Abscissa: dose fentanyl in mg/kg. Ordinate: % mean saline control value for GI transit or respiration. All points show mean ± SEM from 12 mice (6 male, 6 female). Filled points indicate significantly different from saline, *p* < 0.05. The effects of 0.32 and 1.0 mg/kg fentanyl on respiration were not significantly different from each other (N.S.). Center panel and right panel show effects of different fentanyl/naltrexone mixtures composed of 0.32 mg/kg fentanyl + a naltrexone dose determined by the proportion. *Asterisk indicates significantly different from saline (Sal), and ^carat indicates significantly different from 0.32 mg/kg fentanyl alone (0.32 Fent), *p* < 0.05. All bars show mean ± SEM from 12 mice (6 male, 6 female), and points show data from individual mice.

Inhibition of gastrointestinal transit and respiratory depression are two clinically important side effects of MOR agonist analgesics (5, 36). The fentanyl/naltrexone mixture approach was used to assess the role of MOR efficacy as a determinant of these undesirable effects for comparison to the efficacy requirements for antinociception. After saline administration, the mean ± SEM % gastrointestinal transit in the charcoal-meal assay was 84.2 ± 2.4%, and the mean ± SEM minute volume in the plethysmography assay of respiration was 161.4 ± 6.2 ml/min, and *t* tests indicated no significant sex differences in these saline-baseline values. All subsequent data were presented as a percentage of these mean saline values. Figure 9 (left panel) shows that fentanyl administered alone produced a dose-dependent decrease in both gastrointestinal transit [F(4,55) = 75.43, *p* < 0.0001] and respiration [F(5,66) = 34.00, *p* < 0.0001]. A dose of 0.32 mg/kg produced maximal effects in both procedures, eliminating gastrointestinal transit and reducing respiration to a degree (approximately 50%) that was not significantly worsened at higher fentanyl doses (1.0 mg/kg shown in the graph; higher doses of 3.2 and 10 mg/kg tested in a subset of mice but data not shown). Note that 0.32 mg/kg fentanyl also significantly decreased crosses in the locomotor procedure. To examine effects of the mixtures, naltrexone was combined with 0.32 mg/kg fentanyl across a range of proportions from 1:1 to 100:1 fentanyl/naltrexone. Administration of 0.32 mg/kg naltrexone alone did not significantly alter either endpoint (data not shown). Figure 9 right panels show that declining fentanyl proportions produced declining effects on both gastrointestinal transit [F(6,77) = 50.21, *p* < 0.0001] and respiration [F(6,77) = 233.05, *p* < 0.0001]. The mixtures produced significant inhibition of gastrointestinal transit at fentanyl/naltrexone proportions ≥3.2:1 and significant respiratory depression at proportions ≥32:1; however, fentanyl/naltrexone proportions ≤32:1 produced significantly weaker effects on gastrointestinal transit and respiration than fentanyl alone. Insofar as fentanyl/naltrexone mixtures of 18:1, 32:1, and 56:1 produced significant antinociception on one or both endpoints in the assay of pain-depressed behavior, these results show that antinociception can be achieved with intermediate levels of MOR efficacy that produce significant but submaximal inhibition of gastrointestinal transit and respiratory depression. Supplementary Tables S18, S19 shows analysis of these data with the inclusion of sex as an additional variable.

## Discussion

### The model of IP acid-depressed locomotor activity

This study confirms and extends previous findings to indicate that IP acid is effective as a noxious stimulus to produce a pain-related depression of locomotion and other behaviors in rodents (18, 20, 27, 46–48). IP acid models tissue acidosis that occurs in inflammation, traumatic injury, or disease, and it activates peptidergic visceral nociceptors that express proton-sensitive ion channels (e.g., acid-sensing ion channels and transient receptor potential vanilloid 1 channels) (29, 30, 49, 50). Moreover, the peptidergic nociceptors that innervate the rodent peritoneal cavity have relatively high homology to the almost exclusively peptidergic phenotype of human nociceptors, whereas many rodent somatic nociceptors are nonpeptidergic and hence phenotypically more distinct from human nociceptors (50).

Preclinical-to-clinical translation can be addressed not only by using IP acid as the preclinical pain stimulus, but also by using behavioral depression as an endpoint of pain behavior (15, 20, 26). Behavioral depression is a common sign of clinically relevant pain and target of pain treatment, and in preclinical evaluation of candidate analgesics (16, 17), pain-depressed behaviors do not yield false-positive results with treatments that impair behavior (24, 26). This study used a two-compartment activity chamber to enable automated, objective, and quantitative measurement of both horizontal activity (movement counts) and vertical activity (crosses). Notably, the procedure requires no pre-test habituation or training in the apparatus. Moreover, baseline behavior during 15-min sessions was both high and stable across multiple cohorts of mice, and IP acid produced reliable behavioral depression. Taken together, these features of behavioral assessment also support the utility of this procedure for translational, high-throughput, and objective evaluation of treatment effects on pain-related behavioral depression.

### Drug effects on IP acid-induced behavioral depression

**Figure 10:**
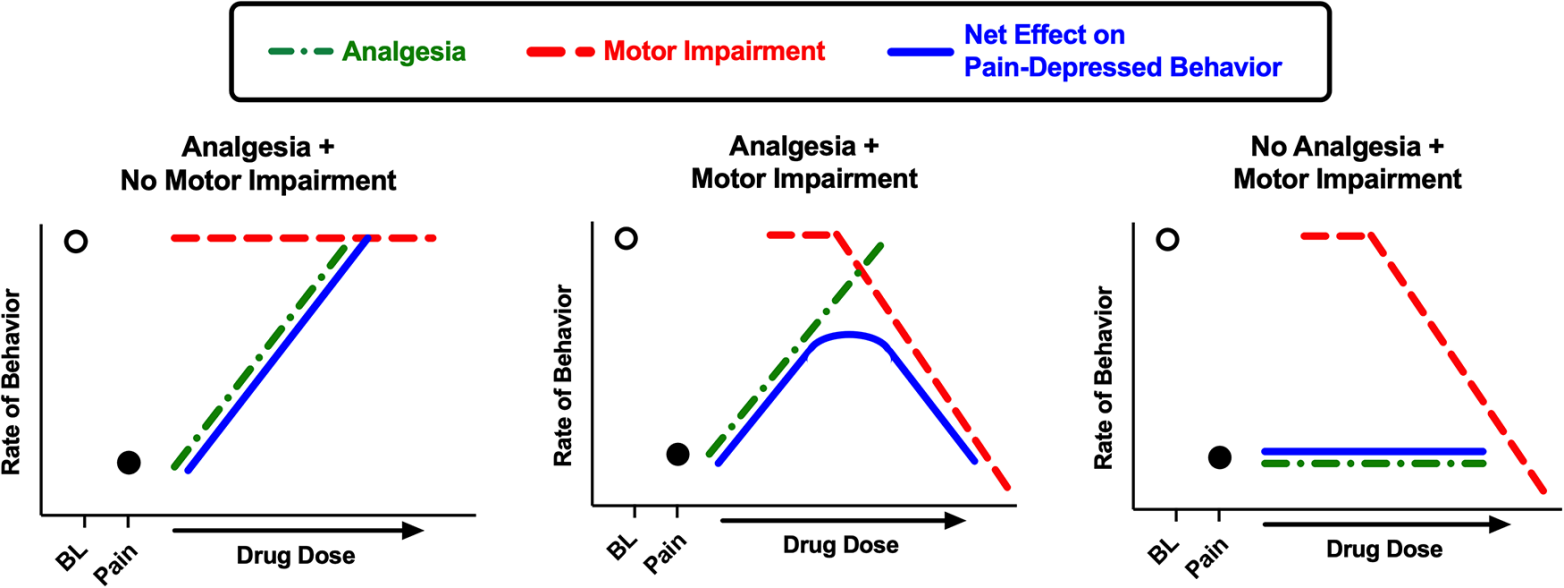
Schematic illustration of drug effects in preclinical assays of pain-depressed behavior. In assays of pain-depressed behavior, the noxious stimulus decreases expression of the measured behavior from high baseline levels (open circle over “BL”) to low levels in the pain state (filled circle over “Pain”). In the present study, IP acid served as the noxious stimulus to decrease crosses and movement counts as measures of locomotor behavior. Drugs can produce analgesic effects that block effects of the noxious stimulus and increase behavior (green dotted line) and/or motor impairment effects that decrease behavior (red dashed line), and these effects are integrated to produce a net change in expression of the pain-depressed behavior (blue solid line). When a drug produces analgesia without motor impairment, then the drug dose-dependently restores behavior back to baseline levels (left panel; e.g. ketoprofen in the present study). When a drug produces both analgesia and motor impairment, then net effects will depend on the degree of separation between analgesic and motor impairing effects, and motor impairment may constrain analgesia-induced restoration of pain-depressed behavior (center panel; e.g. many MOR agonists in the present study). Lastly, if a drug produces no analgesia but does produce motor impairment, then the drug will fail to restore pain-depressed behavior and may exacerbate pain-related behavioral depression (right panel; e.g. diazepam in the present study).

Figure 10 provides a theoretical framework for interpretation of drug effects in this study. As we have described previously (27), the net effects of a drug on pain-depressed behavior depend on an integration of analgesic effects (which attenuate effects of the noxious stimulus and increase behavior) and motor effects (which disrupt and usually decrease ongoing behavior; motor stimulant effects will be discussed below). Notably, motor impairment can oppose and limit the expression of analgesic effects. As a result, a drug is most effective in assays of pain-depressed behavior if it produces analgesia at doses that produce little or no motor impairment.

Our main goal was to examine effects of MOR agonists. To provide a context for interpreting opioid effects, the effects of non-MOR positive and negative controls were also examined. Consistent with its effectiveness to treat many types of clinical pain and to alleviate other examples of IP acid-induced behavioral depression in rodents, the positive control NSAID analgesic ketoprofen fully blocked IP acid-induced depression of both crosses and movement at doses that had no effect on these endpoints when ketoprofen was administered alone (35, 46, 47, 51). By contrast, the negative controls diazepam, U69593, and psilocybin produced dose-dependent behavioral depression when administered alone consistent with other evidence for their motor effects (51–56), and lower doses that did not impair motor function failed to alleviate IP acid-induced behavioral depression. These findings imply that diazepam, U69593, and psilocybin either do not produce analgesia or do so only at doses equal to or above those that produce motor impairment. Amphetamine was effective to alleviate IP acid-induced behavioral depression, but amphetamine also alleviated behavioral depression by LiCl as a non-pain stimulus, suggesting that amphetamine effects may reflect non-selective behavioral stimulation rather than analgesia (56). Overall, then, this procedure differentiated between clinically effective analgesics and several drug types that are not approved for the clinical treatment of acute pain.

MOR agonists and fentanyl/naltrexone mixtures produced efficacy-dependent effects when administered alone or as pretreatments to IP acid. When administered alone, the commonly observed locomotor stimulant effects of MOR agonists (32, 57) were rarely observed because baseline behavior was already high and resistant to further increases (i.e., a ceiling effect; note that amphetamine alone also did not stimulate behavior). However, the higher efficacy MOR agonists fentanyl and morphine did significantly decrease crosses without significantly altering movement. Only the intermediate-efficacy MOR agonist buprenorphine stimulated behavior insofar as it increased crosses at the highest dose tested; however, buprenorphine did not alter movement, and none of the other opioids or mixtures administered alone affected either endpoint. Thus, the experimental design here revealed efficacy-dependent effectiveness of opioids to decrease vertical activity required for crosses without affecting the high rates of horizontal activity reflected in movement. The depression of crosses over the 1-inch barrier by high-efficacy MOR agonists observed here is consistent with efficacy-dependent opioid-induced depression of climbing by mice in chambers that allow vertical locomotion (46, 58).

When administered as a pretreatment to IP acid, optimal relief of pain-depressed behavior was achieved by the intermediate-efficacy MOR agonists (morphine, buprenorphine) and mixtures (56:1, 32:1, 18:1 fentanyl/naltrexone) that retained sufficient MOR efficacy to produce analgesia with little or no motor impairment. The high-efficacy opioid fentanyl alleviated IP acid-induced depression of movement (the endpoint on which fentanyl alone had no effect), but it failed to alleviate IP acid-induced depression of crosses (the endpoint dose-dependently decreased by fentanyl with a steep dose-effect curve). These results suggest that the potency and effectiveness of fentanyl to impair crossing behavior prevented fentanyl from alleviating IP acid-induced depression of crosses. At the other extreme, the lower MOR efficacy single-molecule opioids (nalbuphine, NAQ, naltrexone) and mixture (10:1 fentanyl/naltrexone) lacked sufficient efficacy to either alter locomotion when administered alone or relieve pain-related behavioral depression produced by IP acid. Notably, NAQ is the only MOR agonist tested in the present study that is not available clinically, but it has been evaluated extensively preclinically and has been shown to distribute to brain after systemic administration and produce weak MOR agonist effects (43, 59, 60).

In considering this efficacy dependence of MOR agonist antinociception in the present study, three additional points warrant mention. First, a growing body of evidence suggests that opioid effectiveness in assays of pain-depressed behavior can be influenced by the type of behavior being measured and the sensitivity of that behavior to disruption by opioids administered alone. Thus, we have also investigated MOR agonists with varying efficacies in (a) a rat assay of IP acid-depressed intracranial self-stimulation (ICSS; a positively reinforced operant behavior) (28), and (b) a mouse assay of IP acid-depressed climbing (46). Opioids across a broad efficacy range stimulate ICSS behavior in rats, and opioids across a similarly broad efficacy range are effective to alleviate IP acid-induced ICSS depression (28, 61–63). Conversely, MOR agonists are highly effective to decrease climbing in mice, and MOR agonists and fentanyl/naltrexone mixtures were uniformly ineffective to alleviate IP acid-induced depression of climbing in mice (46, 58). Relative to ICSS in rats and climbing in mice, the present locomotor endpoints in mice displayed an intermediate sensitivity to MOR agonist efficacy. As we have noted in previous studies (27), these findings have implications not only for MOR agonist effects in preclinical assays of pain-depressed behavior, but also for translation to clinical effects of MOR agonists in humans pain patients. Pain states can interfere with a broad range of different behaviors, and opioid analgesic effectiveness to alleviate pain-related behavioral depression may be influenced by both the behavior under investigation and the sensitivity of that behavior to disruption by the opioid.

Second, it should be noted that drug-induced motor-stimulant effects could theoretically produce false-positive antinociception in the present procedure (56). However, drugs stimulated behavior in only two instances in this study (increased crosses after 0.32 mg/kg buprenorphine and 1.0 mg/kg diazepam), and neither of these treatments alleviated IP acid-induced depression of crosses. Moreover, a comparison of drug effects on behavioral depression produced by IP acid vs. IP LiCl provides an additional strategy to evaluate the selectivity of drug effects on pain-related behavioral depression.

Lastly, some limitations of the present study warrant mention. First, the present study did not evaluate the pharmacokinetics of the test drugs or attempt to relate plasma or brain levels to drug effects using pharmacokinetic/pharmacodynamic (PKPD) analysis [e.g., (64, 65)]. Future studies correlating free brain levels of drug with drug effectiveness in behavioral studies would further clarify the relative efficacy of the compounds and efficacy dependence of the effects. Second, validational studies with positive and negative controls in the present study support the proposition that this assay of pain-related behavioral depression may have better preclinical-to-clinical translational predictive validity that conventional assays of pain-stimulated behavior; nonetheless, the translation of results from this procedure to clinical findings in humans remains to be determined.

### Respiratory and gastrointestinal effects

Studies with the fentanyl/naltrexone mixtures also provided evidence for improved safety of analgesic, intermediate-efficacy MOR agonism on respiratory and gastrointestinal side effects (5, 36–39). As expected, fentanyl alone produced a dose-dependent decrease in both gastrointestinal transit and respiration. Fentanyl/naltrexone mixtures effects on both endpoints declined as the fentanyl proportion and associated MOR efficacy declined. Notably, the 32:1 mixture, which relieved IP acid-induced behavioral depression, produced significant gastrointestinal inhibition and respiratory depression, but these effects were significantly attenuated relative to fentanyl alone.

## Conclusion

There were three main findings from this study. First, this procedure generated high and reliable baseline behavioral rates that were reliably depressed by IP acid as an acute visceral noxious stimulus. Second, drugs could be efficiently evaluated for their antinociceptive effectiveness to alleviate this example of pain-related behavioral depression. The positive-control NSAID analgesic ketoprofen and several MOR agonists and mixtures produced antinociception, but a series of non-analgesic negative controls did not. MOR agonist effects were efficacy dependent, with optimal effects by intermediate-efficacy opioids, and unlike the stimulant amphetamine, MOR agonists selectively alleviated behavioral depression produced by IP acid but not by IP LiCl. Lastly, studies with fentanyl/naltrexone mixtures indicated that effective relief of pain-depressed behavior could be achieved with significant but submaximal levels of gastrointestinal transit inhibition and respiratory depression. Overall, these findings validate a novel procedure for evaluating candidate analgesic effects on pain-related behavioral depression in mice and support continued research with low- to intermediate-efficacy MOR agonists as a strategy to retain analgesic effectiveness with improved safety.

## Data Availability

The original contributions presented in the study are included in the article/**Supplementary Material**, further inquiries can be directed to the corresponding author.
