# Supplementary material for: Role of mu opioid receptor (MOR) agonist efficacy as a determinant of opioid antinociception in a novel assay of pain-depressed behavior in female and male mice
Source: Front Pain Res (Lausanne). 2023 Oct 11;4:1281698. doi: 10.3389/fpain.2023.1281698 (PMC10598607; doi:10.3389/fpain.2023.1281698)
Supplement: Supplementary file 1 [file Table1.docx]

**Supplemental Material For**

**Role of Mu Opioid Receptor (MOR) Agonist Efficacy**

**as a Determinant of Opioid Antinociception**

**in a Novel Assay of Pain-Depressed Behavior in Female and Male Mice**

Negus SS^a^, Akbarali HI, Kang M, Lee YK^a^, Marsh SA^a^, Santos EJ^a^, Zhang Y^b^

^a^Department of Pharmacology & Toxicology, School of Medicine,

Virginia Commonwealth University, Richmond, VA

^b^Department of Medicinal Chemistry, School of Pharmacy,

Virginia Commonwealth University, Richmond VA

**Supplemental Figure 1.** Results of parametric studies to evaluate the effects of session length (top panels) and barrier height between the compartments (bottom panels). The top panels show Crosses (left), Movement Counts (center), and Bias (right) during a 2-hour session with no barrier in the doorway between compartments. Each point shows mean ± SEM from a single group of 12 mice (6 male, 6 female). There was a significant effect of time on Crosses [F(2.45, 26.92)=28.51, p<0.0001] and Movement [F(2.44, 26.90)=45.68, p<0.0001]. Asterisks (***) indicate that both Crosses and Movement were higher during the first 15 min than during any 15-min period during the rest of the session, p<0.001. By contrast, Bias did not change significantly over the course of the session. When sex was included as an additional variable in a two-way ANOVA, there was no a main effect of Sex or a Sex x Time interaction for any endpoint. Because a major goal was to examine expression and treatment of pain-related behavioral depression, all subsequent studies used a 15-min session time when baseline crosses and movement counts in the absence of a pain stimulus were highest.

The bottom panels show Crosses (left), Movement Counts (center), and Bias (right) during 15-min sessions when the height of doorway barrier was varied from 0 to 1.5 inches. Each bar shows mean ± SEM from a separate group of 12 mice (6 male, 6 female), and points show data from individual mice. There was a significant effect of barrier height on Crosses [F(3,43)=58.34, p<0.0001], and asterisks (**) indicate that Crosses were lower at all barrier heights than when there was no barrier, p<0.001. By contrast, barrier height had no effect on Movement Counts or Bias. When sex was included as a variable in two-way ANOVA of barrier-height effects, there was no main effect of Sex or Sex x Height interaction on crosses. Subsequent studies used a 1-inch barrier height because it struck a compromise between impeding crosses while allowing a sufficient number of remaining baseline crosses to detect further pain-related depression.


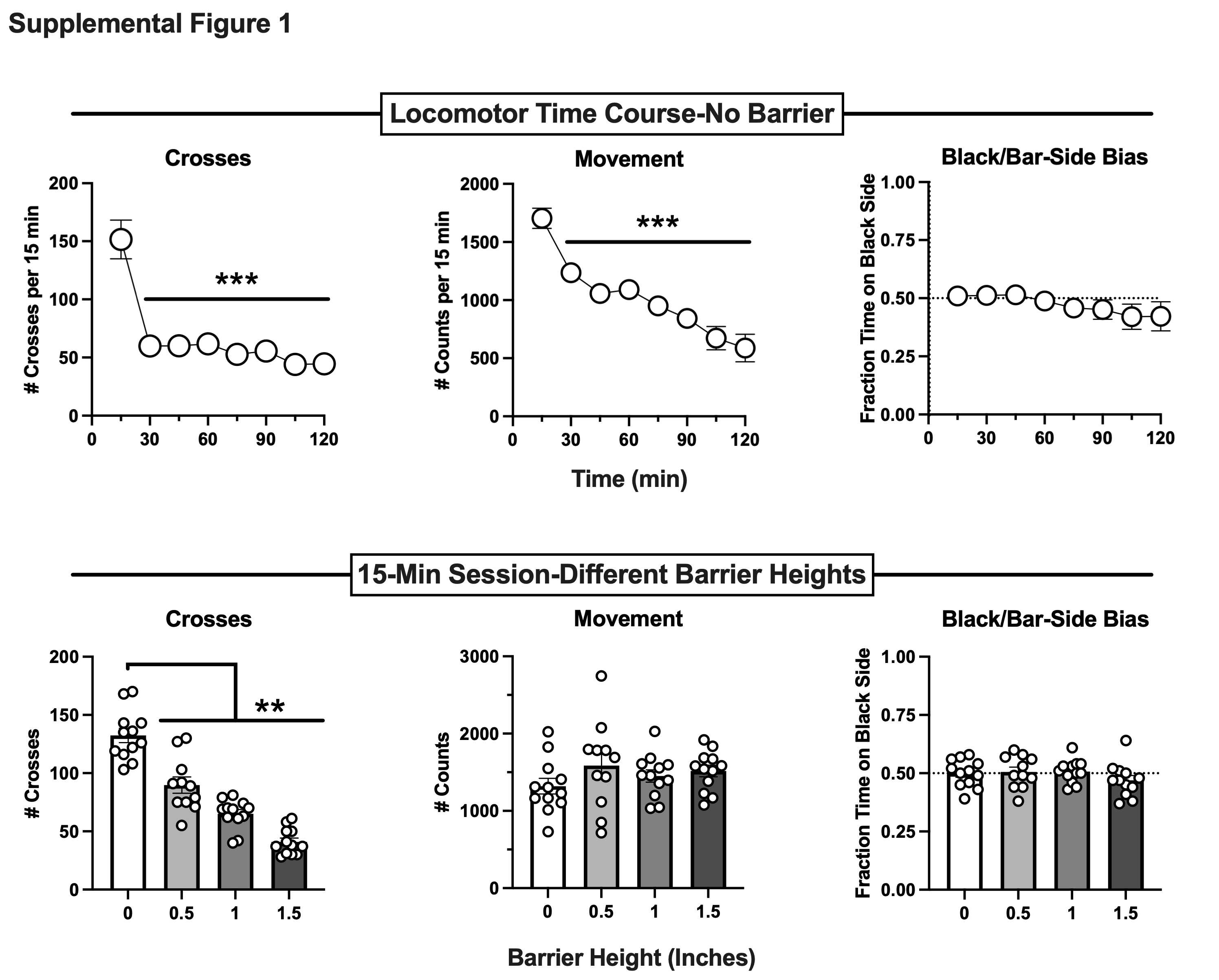


**Supplemental Figure 2.** Effects of sex on baseline activity measures in the absence of IP acid (No Inj=no injection) or in the presence of 0.56% IP acid across all 15 cohorts of mice used to evaluate test drugs. Top panel shows data for Crosses (left), Movement Counts (center), and Bias for the compartment with black walls and bar floor (right). Each bar shows mean±SEM in 90 mice, and points show data from individual mice. Lower panel shows 2-way ANOVA results for each endpoint. Significant p values are shown in bold in the right column.


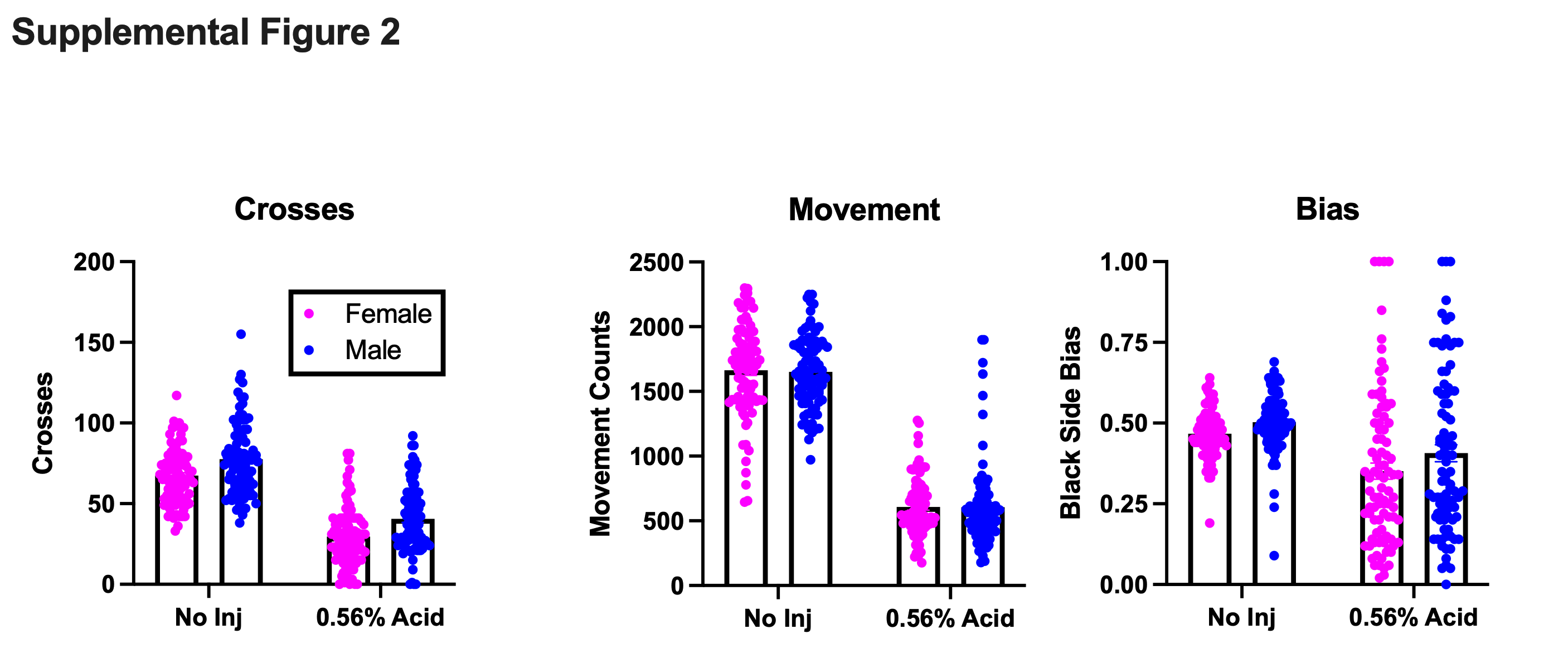


| **Endpoint** | **Factor** | **F value** | **P value** |
| --- | --- | --- | --- |
| Crosses | Main Effect of Acid | F(1,356) = 318.00 | **p<0.0001** |
|  | Main Effect of Sex | F(1,356) = 25.77 | **p<0.0001** |
|  | Acid x Sex Interaction | F(1,356) = 0.048 | p=0.8258 |
| Movement Counts | Main Effect of Acid | F(1,356) = 1071.00 | **p<0.0001** |
|  | Main Effect of Sex | F(1,356) = 0.05 | p=0.8190 |
|  | Acid x Sex Interaction | F(1,356) = 0.02 | p=0.8800 |
| Bias | Main Effect of Acid | F(1,356) = 30.56 | **p<0.0001** |
|  | Main Effect of Sex | F(1,356) = 5.77 | **p=0.0168** |
|  | Acid x Sex Interaction | F(1,356) = 0.26 | p=0.6100 |

Supplementary Table 1: Power analysis for ketoprofen alone and as a pretreatment to IP acid

| **TX** | **Measure** | **Factor** | **F statistic, p value** | **Cohen’s Effect Size (Cohen’s F)** | **Current Power** | **Sample Size: Power≥0.8** |
| --- | --- | --- | --- | --- | --- | --- |
| **Drug**  **Alone** | Crosses | Dose | F (4, 50) = 1.72; P=0.1612 | 0.371 | 0.573 | 95 |
|  |  | Sex | F (1, 50) = 0.32; P=0.5753 | 0.080 | 0.093 | >100 |
|  |  | Dose x Sex | F (4, 50) = 1.69; P=0.1661 | 0.068 | 0.063 | >100 |
|  | Movement | Dose | F (4, 50) = 4.18; P=0.0054 | 0.578 | 0.946 | 43 |
|  |  | Sex | F (1, 50) = 0.79; P=0.3793 | 0.125 | 0.159 | >100 |
|  |  | Dose x Sex | F (4, 50) = 0.56; P=0.6948 | 0.211 | 0.203 | >100 |
| **Drug +**  **IP Acid** | Crosses | Dose | F (4, 50) = 4.92; p=0.0020 | 0.628 | 0.975 | 36 |
|  |  | Sex | F (1, 50) = 0.05; p=0.8255 | 0.031 | 0.057 | >100 |
|  |  | Dose x Sex | F (4, 50) = 1.00; p=0.4145 | 0.283 | 0.350 | >100 |
|  | Movement | Dose | F (4, 50) = 21.32; p<0.0001 | 1.306 | 1 | 16 |
|  |  | Sex | F (1, 50) = 7.98; p=0.0068 | 0.399 | 0.859 | 51 |
|  |  | Dose x Sex | F (4, 50) = 1.77; p=0.1490 | 0.377 | 0.589 | 90 |

Supplementary Table 2: Power analysis for fentanyl alone and as a pretreatment to IP acid

| **TX** | **Measure** | **Factor** | **F statistic, p value** | **Cohen’s Effect Size (Cohen’s F)** | **Current Power** | **Sample Size: Power≥0.8** |
| --- | --- | --- | --- | --- | --- | --- |
| **Drug**  **Alone** | Crosses | Dose | F (4, 50) = 10.65; P<0.0001 | 0.923 | 0.999 | 22 |
|  |  | Sex | F (1, 50) = 0.14; P=0.7116 | 0.053 | 0.069 | >100 |
|  |  | Dose x Sex | F (4, 50) = 0.64; P=0.6393 | 0.226 | 0.228 | >100 |
|  | Movement | Dose | F (4, 50) = 1.24; P=0.3046 | 0.315 | 0.429 | >100 |
|  |  | Sex | F (1, 50) = 0.0009; P=0.9755 | 0.004 | 0.050 | >100 |
|  |  | Dose x Sex | F (4, 50) = 0.82; P=0.5219 | 0.255 | 0.287 | >100 |
| **Drug +**  **IP Acid** | Crosses | Dose | F (4, 50) = 1.40; P=0.2486 | 0.334 | 0.478 | >100 |
|  |  | Sex | F (1, 50) = 0.47; P=0.4979 | 0.097 | 0.114 | >100 |
|  |  | Dose x Sex | F (4, 50) = 1.51; P=0.2139 | 0.347 | 0.512 | >100 |
|  | Movement | Dose | F (4, 50) = 7.59; P<0.0001 | 0.779 | 0.999 | 27 |
|  |  | Sex | F (1, 50) = 2.06; P=0.1578 | 0.203 | 0.338 | >100 |
|  |  | Dose x Sex | F (4, 50) = 0.37; P=0.8266 | 0.173 | 0.146 | >100 |

Supplementary Table 3: Power analysis for morphine alone and as a pretreatment to IP acid

| **TX** | **Measure** | **Factor** | **F statistic, p value** | **Cohen’s Effect Size (Cohen’s F)** | **Current Power** | **Sample Size: Power≥0.8** |
| --- | --- | --- | --- | --- | --- | --- |
| **Drug**  **Alone** | Crosses | Dose | F (4, 49) = 4.87; P=0.0022 | 0.631 | 0.974 | 36 |
|  |  | Sex | F (1, 49) = 0.19; P=0.6687 | 0.062 | 0.075 | >100 |
|  |  | Dose x Sex | F (4, 49) = 0.32; P=0.8642 | 0.161 | 0.131 | >100 |
|  | Movement | Dose | F (4, 49) = 3.04; P=0.0258 | 1.744 | 1 | 14 |
|  |  | Sex | F (1, 49) = 0.04; P=0.8386 | 0.205 | 0.338 | >100 |
|  |  | Dose x Sex | F (4, 49) = 0.52; P=0.7226 | 0.720 | 0.995 | 30 |
| **Drug +**  **IP Acid** | Crosses | Dose | F (5, 60) = 3.27; P=0.0113 | 0.522 | 0.925 | 55 |
|  |  | Sex | F (1, 60) = 0.08; P=0.7750 | 0.037 | 0.061 | >100 |
|  |  | Dose x Sex | F (5, 60) = 2.24; P=0.0620 | 0.432 | 0.779 | 77 |
|  | Movement | Dose | F (5, 60) = 3.26: P=0.0114 | 0.521 | 0.925 | 55 |
|  |  | Sex | F (1, 60) = 0.124; P=0.7263 | 0.045 | 0.067 | >100 |
|  |  | Dose x Sex | F (5, 60) = 1.14; P=0.3481 | 0.309 | 0.451 | >100 |

Supplementary Table 4: Power analysis for buprenorphine alone and as a pretreatment to IP acid

| **TX** | **Measure** | **Factor** | **F statistic, p value** | **Cohen’s Effect Size (Cohen’s F)** | **Current Power** | **Sample Size: Power≥0.8** |
| --- | --- | --- | --- | --- | --- | --- |
| **Drug**  **Alone** | Crosses | Dose | F (4, 50) = 3.57; P=0.0123 | 0.534 | 0.904 | 49 |
|  |  | Sex | F (1, 50) = 1.83; P=0.1822 | 0.191 | 0.307 | >100 |
|  |  | Dose x Sex | F (4, 50) = 1.28; P=0.2890 | 0.321 | 0.442 | >100 |
|  | Movement | Dose | F (4, 50) = 0.52; P=0.7235 | 0.203 | 0.190 | >100 |
|  |  | Sex | F (1, 50) = 0.97; P=0.3303 | 0.139 | 0.184 | >100 |
|  |  | Dose x Sex | F (4, 50) = 2.55; P=0.0504 | 0.452 | 0.768 | 66 |
| **Drug +**  **IP Acid** | Crosses | Dose | F (6, 74) = 2.86; P=0.0147 | 0.481 | 0.923 | 68 |
|  |  | Sex | F (1, 74) = 1.46; P=0.2314 | 0.140 | 0.255 | >100 |
|  |  | Dose x Sex | F (6, 74) = 1.30; P=0.2689 | 0.324 | 0.562 | >100 |
|  | Movement | Dose | F (6, 74) = 7.18; P<0.0001 | 0.763 | 0.999 | 33 |
|  |  | Sex | F (1, 74) = 0.05; P=0.8255 | 0.026 | 0.057 | >100 |
|  |  | Dose x Sex | F (6, 74) = 1.88; P=0.0964 | 0.390 | 0.751 | 99 |

Supplementary Table 5: Power analysis for nalbuphine alone and as a pretreatment to IP acid

| **TX** | **Measure** | **Factor** | **F statistic, p value** | **Cohen’s Effect Size (Cohen’s F)** | **Current Power** | **Sample Size: Power≥0.8** |
| --- | --- | --- | --- | --- | --- | --- |
| **Drug**  **Alone** | Crosses | Dose | F (4, 50) = 1.75; P=0.1537 | 0.374 | 0.583 | 93 |
|  |  | Sex | F (1, 50) = 0.91; P=0.3459 | 0.135 | 0.176 | >100 |
|  |  | Dose x Sex | F (4, 50) = 0.77; P=0.5499 | 0.248 | 0.272 | >100 |
|  | Movement | Dose | F (4, 50) = 3.40; P=0.0156 | 0.521 | 0.887 | 51 |
|  |  | Sex | F (1, 50) = 3.31; P=0.0749 | 0.256 | 0.498 | >100 |
|  |  | Dose x Sex | F (4, 50) = 1.05; P=0.3894 | 0.290 | 0.366 | >100 |
| **Drug +**  **IP Acid** | Crosses | Dose | F (4, 50) = 0.48;  P=0.7541 | 0.195 | 0.177 | >100 |
|  |  | Sex | F (1, 50) = 3.21; P=0.0791 | 0.254 | 0.486 | >100 |
|  |  | Dose x Sex | F (4, 50) = 0.73; P=0.5779 | 0.241 | 0.256 | >100 |
|  | Movement | Dose | F (4, 50) = 0.97; P=0.4327 | 0.279 | 0.338 | >100 |
|  |  | Sex | F (1, 50) = 2.56; P=0.1162 | 0.226 | 0.404 | >100 |
|  |  | Dose x Sex | F (4, 50) = 0.89; P=0.4752 | 0.267 | 0.313 | >100 |

Supplementary Table 6: Power analysis for NAQ alone and as a pretreatment to IP acid

| **TX** | **Measure** | **Factor** | **F statistic, p value** | **Cohen’s Effect Size (Cohen’s F)** | **Current Power** | **Sample Size: Power≥0.8** |
| --- | --- | --- | --- | --- | --- | --- |
| **Drug**  **Alone** | Crosses | Dose | F (4, 50) = 0.73; P=0.5779 | 0.241 | 0.258 | >100 |
|  |  | Sex | F (1, 50) = 1.20; P=0.2785 | 0.155 | 0.218 | >100 |
|  |  | Dose x Sex | F (4, 50) = 1.17; P=0.3372 | 0.305 | 0.403 | >100 |
|  | Movement | Dose | F (4, 50) = 0.63; P=0.6463 | 0.224 | 0.225 | >100 |
|  |  | Sex | F (1, 50) = 0.17; P=0.6818 | 0.058 | 0.073 | >100 |
|  |  | Dose x Sex | F (4, 50) = 0.45; P=0.7732 | 0.189 | 0.169 | >100 |
| **Drug +**  **IP Acid** | Crosses | Dose | F (4, 50) = 0.16; P=0.9587 | 0.112 | 0.087 | >100 |
|  |  | Sex | F (1, 50) = 17.18; P=0.0001 | 0.586 | 0.994 | 27 |
|  |  | Dose x Sex | F (4, 50) = 0.99; P=0.4199 | 0.282 | 0.346 | >100 |
|  | Movement | Dose | F (4, 50) = 0.98; P=0.4257 | 0.280 | 0.343 | >100 |
|  |  | Sex | F (1, 50) = 1.01; P=0.3187 | 0.143 | 0.191 | >100 |
|  |  | Dose x Sex | F (4, 50) = 2.85; P=0.0334 | 0.477 | 0.818 | 60 |

Supplementary Table 7: Power analysis for naltrexone alone and as a pretreatment to IP acid

| **TX** | **Measure** | **Factor** | **F statistic, p value** | **Cohen’s Effect Size (Cohen’s F)** | **Current Power** | **Sample Size: Power≥0.8** |
| --- | --- | --- | --- | --- | --- | --- |
| **Drug**  **Alone** | Crosses | Dose | F (1, 20) = 0.11; P=0.7471 | 0.073 | 0.063 | >100 |
|  |  | Sex | F (1, 20) = 13.34;  P=0.0016 | 0.817 | 0.968 | 15 |
|  |  | Dose x Sex | F (1, 20) = 0.13; P=0.7194 | 0.082 | 0.067 | >100 |
|  | Movement | Dose | F (1, 20) = 0.43; P=0.5194 | 0.147 | 0.105 | >100 |
|  |  | Sex | F (1, 20) = 2.88; P=0.1054 | 0.379 | 0.424 | 59 |
|  |  | Dose x Sex | F (1, 20) = 0.42; P=0.5226 | 0.146 | 0.104 | >100 |
| **Drug +**  **IP Acid** | Crosses | Dose | F (1, 20) = 0.45; P=0.5120 | 0.149 | 0.107 | >100 |
|  |  | Sex | F (1, 20) = 1.63; P=0.2165 | 0.285 | 0.265 | >100 |
|  |  | Dose x Sex | F (1, 20) = 0.02; P=0.9004 | 0.028 | 0.052 | >100 |
|  | Movement | Dose | F (1, 20) = 0.06; P=0.8020 | 0.057 | 0.058 | >100 |
|  |  | Sex | F (1, 20) = 0.23; P=0.6304 | 0.109 | 0.080 | >100 |
|  |  | Dose x Sex | F (1, 20) = 0.34; P=0.5676 | 0.130 | 0.093 | >100 |

Supplementary Table 8: Power analysis for 56:1 Fent/NTX alone and as a pretreatment to IP acid

| **TX** | **Measure** | **Factor** | **F statistic, p value** | **Cohen’s Effect Size (Cohen’s F)** | **Current Power** | **Sample Size: Power≥0.8** |
| --- | --- | --- | --- | --- | --- | --- |
| **Drug**  **Alone** | Crosses | Dose | F (4, 50) = 0.12; P=0.9743 | 0.098 | 0.078 | >100 |
|  |  | Sex | F (1, 50) = 2.19; P=0.1455 | 0.209 | 0.355 | >100 |
|  |  | Dose x Sex | F (4, 50) = 0.64; P=0.6370 | 0.226 | 0.229 | >100 |
|  | Movement | Dose | F (4, 50) = 0.26; P=0.9014 | 0.145 | 0.114 | >100 |
|  |  | Sex | F (1, 50) = 2.78; P=0.1020 | 0.236 | 0.433 | >100 |
|  |  | Dose x Sex | F (4, 50) = 0.19; P=0.9392 | 0.125 | 0.097 | >100 |
| **Drug +**  **IP Acid** | Crosses | Dose | F (4, 50) = 3.72; P=0.0100 | 0.545 | 0.916 | 47 |
|  |  | Sex | F (1, 50) = 2.85; P=0.0974 | 0.239 | 0.442 | >100 |
|  |  | Dose x Sex | F (4, 50) = 0.49; P=0.7432 | 0.198 | 0.182 | >100 |
|  | Movement | Dose | F (4, 50) = 14.83; P<0.0001 | 1.089 | 0.999 | 19 |
|  |  | Sex | F (1, 50) = 4.19; P=0.0459 | 0.290 | 0.595 | 99 |
|  |  | Dose x Sex | F (4, 50) = 0.42; P=0.7910 | 0.184 | 0.161 | >100 |

Supplementary Table 9: Power analysis for 32:1 Fent/NTX alone and as a pretreatment to IP acid

| **TX** | **Measure** | **Factor** | **F statistic, p value** | **Cohen’s Effect Size (Cohen’s F)** | **Current Power** | **Sample Size: Power≥0.8** |
| --- | --- | --- | --- | --- | --- | --- |
| **Drug**  **Alone** | Crosses | Dose | F (4, 50) = 0.15; P=0.9627 | 0.109 | 0.085 | >100 |
|  |  | Sex | F (1, 50) = 2.27; P=0.1380 | 0.213 | 0.367 | >100 |
|  |  | Dose x Sex | F (4, 50) = 1.10; P=0.3657 | 0.297 | 0.383 | >100 |
|  | Movement | Dose | F (4, 50) = 0.38; P=0.8201 | 0.175 | 0.149 | >100 |
|  |  | Sex | F (1, 50) = 3.41; P=0.0708 | 0.262 | 0.510 | >100 |
|  |  | Dose x Sex | F (4, 50) = 1.54; P=0.2039 | 0.352 | 0.523 | >100 |
| **Drug +**  **IP Acid** | Crosses | Dose | F (4, 50) = 3.15; P=0.0218 | 0.502 | 0.859 | 54 |
|  |  | Sex | F (1, 50) = 2.52; P=0.1190 | 0.224 | 0.399 | >100 |
|  |  | Dose x Sex | F (4, 50) = 0.99; P=0.4197 | 0.282 | 0.347 | >100 |
|  | Movement | Dose | F (4, 50) = 6.66; P=0.0002 | 0.731 | 0.996 | 30 |
|  |  | Sex | F (1, 50) = 1.55; P=0.2184 | 0.176 | 0.268 | >100 |
|  |  | Dose x Sex | F (4, 50) = 1.19; P=0.3279 | 0.308 | 0.411 | >100 |

Supplementary Table 10: Power analysis for 18:1 Fent/NTX alone and as a pretreatment to IP acid

| **TX** | **Measure** | **Factor** | **F statistic, p value** | **Cohen’s Effect Size (Cohen’s F)** | **Current Power** | **Sample Size: Power≥0.8** |
| --- | --- | --- | --- | --- | --- | --- |
| **Drug**  **Alone** | Crosses | Dose | F (4, 50) = 0.41; P=0.8030 | 0.181 | 0.156 | >100 |
|  |  | Sex | F (1, 50) = 0.09; P=0.7647 | 0.043 | 0.062 | >100 |
|  |  | Dose x Sex | F (4, 50) = 0.94; P=0.4495 | 0.274 | 0.328 | >100 |
|  | Movement | Dose | F (4, 50) = 0.30; P=0.8780 | 0.154 | 0.125 | >100 |
|  |  | Sex | F (1, 50) = 0.20; P=0.6544 | 0.064 | 0.077 | >100 |
|  |  | Dose x Sex | F (4, 50) = 0.68; P=0.6119 | 0.233 | 0.241 | >100 |
| **Drug +**  **IP Acid** | Crosses | Dose | F (4, 50) = 2.40; P=0.0624 | 0.438 | 0.739 | 69 |
|  |  | Sex | F (1, 50) = 0.71; P=0.4040 | 0.119 | 0.148 | >100 |
|  |  | Dose x Sex | F (4, 50) = 2.51; P=0.0538 | 0.448 | 0.760 | 67 |
|  | Movement | Dose | F (4, 50) = 3.53; P=0.0130 | 0.531 | 0.899 | 49 |
|  |  | Sex | F (1, 50) = 0.145; P=0.7053 | 0.054 | 0.069 | >100 |
|  |  | Dose x Sex | F (4, 50) = 1.55; P=0.2023 | 0.352 | 0.525 | >100 |

Supplementary Table 11: Power analysis for 10:1 Fent/NTX alone and as a pretreatment to IP acid

| **TX** | **Measure** | **Factor** | **F statistic, p value** | **Cohen’s Effect Size (Cohen’s F)** | **Current Power** | **Sample Size: Power≥0.8** |
| --- | --- | --- | --- | --- | --- | --- |
| **Drug**  **Alone** | Crosses | Dose | F (4, 50) = 0.23; P=0.9195 | 0.136 | 0.106 | >100 |
|  |  | Sex | F (1, 50) = 0.37; P=0.5458 | 0.086 | 0.100 | >100 |
|  |  | Dose x Sex | F (4, 50) = 0.09; P=0.9861 | 0.083 | 0.070 | >100 |
|  | Movement | Dose | F (4, 50) = 0.50; P=0.7326 | 0.201 | 0.186 | >100 |
|  |  | Sex | F (1, 50) = 1.48; P=0.2302 | 0.172 | 0.257 | >100 |
|  |  | Dose x Sex | F (4, 50) = 2.67; P=0.0430 | 0.462 | 0.789 | 63 |
| **Drug +**  **IP Acid** | Crosses | Dose | F (4, 50) = 0.75; P=0.5619 | 0.245 | 0.266 | >100 |
|  |  | Sex | F (1, 50) = 0.15; P=0.6976 | 0.055 | 0.070 | >100 |
|  |  | Dose x Sex | F (4, 50) = 0.17; P=0.9551 | 0.115 | 0.089 | >100 |
|  | Movement | Dose | F (4, 50) = 1.39; P=0.2479 | 0.335 | 0.478 | >100 |
|  |  | Sex | F (1, 50) = 0.24; P=0.6268 | 0.069 | 0.082 | >100 |
|  |  | Dose x Sex | F (4, 50) = 0.68; P=0.6099 | 0.233 | 0.242 | >100 |

Supplementary Table 12: Power analysis for diazepam alone and as a pretreatment to IP acid

| **TX** | **Measure** | **Factor** | **F statistic, p value** | **Cohen’s Effect Size (Cohen’s F)** | **Current Power** | **Sample Size: Power≥0.8** |
| --- | --- | --- | --- | --- | --- | --- |
| **Drug**  **Alone** | Crosses | Dose | F (5, 60) = 12.71; P<0.0001 | 1.03 | 1 | 22 |
|  |  | Sex | F (1, 60) = 0.30; P=0.5880 | 0.070 | 0.090 | >100 |
|  |  | Dose x Sex | F (5, 60) = 0.87; P=0.5047 | 0.270 | 0.348 | >100 |
|  | Movement | Dose | F (5, 60) = 21.86; P<0.0001 | 1.350 | 1 | 18 |
|  |  | Sex | F (1, 60) = 4.44; P=0.0392 | 0.272 | 0.623 | >100 |
|  |  | Dose x Sex | F (5, 60) = 2.11; P=0.0771 | 0.419 | 0.749 | 81 |
| **Drug +**  **IP Acid** | Crosses | Dose | F (5, 60) = 4.08; P=0.0030 | 0.583 | 0.972 | 46 |
|  |  | Sex | F (1, 60) = 0.86; P=0.3586 | 0.119 | 0.169 | >100 |
|  |  | Dose x Sex | F (5, 60) = 0.63; P=0.6788 | 0.229 | 0.253 | >100 |
|  | Movement | Dose | F (5, 60) = 10.51; P<0.0001 | 0.936 | 0.999 | 24 |
|  |  | Sex | F (1, 60) = 1.68; P=0.2003 | 0.167 | 0.287 | >100 |
|  |  | Dose x Sex | F (5, 60) = 0.82; P=0.5427 | 0.261 | 0.326 | >100 |

Supplementary Table 13: Power analysis for U69593 alone and as a pretreatment to IP acid

| **TX** | **Measure** | **Factor** | **F statistic, p value** | **Cohen’s Effect Size (Cohen’s F)** | **Current Power** | **Sample Size: Power≥0.8** |
| --- | --- | --- | --- | --- | --- | --- |
| **Drug**  **Alone** | Crosses | Dose | F (3, 40) = 8.89; P=0.0001 | 0.816 | 0.998 | 22 |
|  |  | Sex | F (1, 40) = 0.24; P=0.6254 | 0.078 | 0.082 | >100 |
|  |  | Dose x Sex | F (3, 40) = 3.88; P=0.0158 | 0.540 | 0.861 | 43 |
|  | Movement | Dose | F (3, 40) = 21.54; P<0.0001 | 1.271 | 1 | 14 |
|  |  | Sex | F (1, 40) = 28.10; P<0.0001 | 0.838 | 0.999 | 16 |
|  |  | Dose x Sex | F (3, 40) = 0.91; P=0.4438 | 0.262 | 0.273 | >100 |
| **Drug +**  **IP Acid** | Crosses | Dose | F (4, 50) = 0.08; P=0.9884 | 0.080 | 0.068 | >100 |
|  |  | Sex | F (1, 50) = 4.02; P=0.0505 | 0.283 | 0.577 | >100 |
|  |  | Dose x Sex | F (4, 50) = 0.41; P=0.7951 | 0.183 | 0.160 | >100 |
|  | Movement | Dose | F (4, 50) = 0.39; P=0.8121 | 0.177 | 0.152 | >100 |
|  |  | Sex | F (1, 50) = 0.35; P=0.5555 | 0.084 | 0.098 | >100 |
|  |  | Dose x Sex | F (4, 50) = 1.05; P=0.3904 | 0.290 | 0.366 | >100 |

Supplementary Table 14: Power analysis for psilocybin alone and as a pretreatment to IP acid

| **TX** | **Measure** | **Factor** | **F statistic, p value** | **Cohen’s Effect Size (Cohen’s F)** | **Current Power** | **Sample Size: Power≥0.8** |
| --- | --- | --- | --- | --- | --- | --- |
| **Drug**  **Alone** | Crosses | Dose | F (4, 50) = 2.46; P=0.0571 | 0.444 | 0.752 | >100 |
|  |  | Sex | F (1, 50) = 0.69; P=0.4103 | 0.117 | 0.145 | >100 |
|  |  | Dose x Sex | F (4, 50) = 1.16; P=0.3399 | 0.305 | 0.401 | >100 |
|  | Movement | Dose | F (4, 50) = 13.67; P<0.0001 | 1.046 | 0.999 | 19 |
|  |  | Sex | F (1, 50) = 7.26; P=0.0096 | 0.381 | 0.825 | 58 |
|  |  | Dose x Sex | F (4, 50) = 1.46; P=0.2292 | 0.341 | 0.497 | >100 |
| **Drug +**  **IP Acid** | Crosses | Dose | F (5, 60) = 1.19; P=0.3235 | 0.315 | 0.470 | >100 |
|  |  | Sex | F (1, 60) = 3.11; P=0.0828 | 0.2278 | 0.477 | >100 |
|  |  | Dose x Sex | F (5, 60) = 0.90; P=0.4880 | 0.274 | 0.358 | >100 |
|  | Movement | Dose | F (5, 60) = 1.70; P=0.1482 | 0.377 | 0.641 | 99 |
|  |  | Sex | F (1, 60) = 0.94; P=0.3358 | 0.125 | 0.182 | >100 |
|  |  | Dose x Sex | F (5, 60) = 0.26; P=0.9338 | 0.147 | 0.122 | >100 |

Supplementary Table 15: Power analysis for amphetamine alone and as a pretreatment to IP acid

| **TX** | **Measure** | **Factor** | **F statistic, p value** | **Cohen’s Effect Size (Cohen’s F)** | **Current Power** | **Sample Size: Power≥0.8** |
| --- | --- | --- | --- | --- | --- | --- |
| **Drug**  **Alone** | Crosses | Dose | F (4, 50) = 2.79; P=0.0363 | 0.473 | 0.809 | 61 |
|  |  | Sex | F (1, 50) = 2.07; P=0.1569 | 0.203 | 0.339 | >100 |
|  |  | Dose x Sex | F (4, 50) = 1.88; P=0.1295 | 0.387 | 0.617 | 87 |
|  | Movement | Dose | F (4, 50) = 1.76; P=0.1527 | 0.375 | 0.584 | 93 |
|  |  | Sex | F (1, 50) = 1.45; P=0.2344 | 0.170 | 0.253 | >100 |
|  |  | Dose x Sex | F (4, 50) = 0.90; P=0.4718 | 0.268 | 0.342 | >100 |
| **Drug +**  **IP Acid** | Crosses | Dose | F (3, 40) = 6.42; P=0.0012 | 0.694 | 0.980 | 29 |
|  |  | Sex | F (1, 40) = 0.10; P=0.7571 | 0.049 | 0.063 | >100 |
|  |  | Dose x Sex | F (3, 40) = 0.32; P=0.8120 | 0.155 | 0.119 | >100 |
|  | Movement | Dose | F (3, 40) = 20.85; P<0.0001 | 1.251 | 1 | 14 |
|  |  | Sex | F (1, 40) = 1.03; P=0.3154 | 0.161 | 0.193 | >100 |
|  |  | Dose x Sex | F (3, 40) = 2.33; P=0.0889 | 0.418 | 0.630 | 69 |

**Supplemental Table 16.** Two-way Dose x Sex ANOVA results for effects of lithium chloride (LiCl) administered alone on Crosses and Movement Counts (Figure 8, top panels). Significant p values are shown in bold in the right column. There was a significant main effect of Sex on Movement Counts, with higher values in females than in males. However, the lack of a Dose x Sex interaction indicates that there was not a sex difference in LiCl effects on movement.

| **Endpoint** | **Factor** | **F value** | **P value** |
| --- | --- | --- | --- |
| Crosses | Main Effect of LiCl Dose | F(3,40) = 9.10 | **p<0.0001** |
|  | Main Effect of Sex | F(1,40) = 2.43 | p=0.1266 |
|  | LiCl x Sex Interaction | F(3,40) = 0.12 | p=0.9478 |
| Movement Counts | Main Effect of LiCl Dose | F(3,40) = 24.79 | **p<0.0001** |
|  | Main Effect of Sex | F(1,40) = 6.37 | **p=0.0157** |
|  | LiCl x Sex Interaction | F(3,40) = 0.29 | p=0.8327 |

**Supplemental Table 17.** Two-way Test Drug x Sex ANOVA results for effects of test drugs administered as a pretreatment to LiCl on Crosses and Movement Counts (Figure 8, bottom panels). Significant p values are shown in bold in the right column. There was not a significant main effect of Sex or Test Drug x Sex interaction on either endpoint.

| **Endpoint** | **Factor** | **F value** | **P value** |
| --- | --- | --- | --- |
| Crosses | Main Effect of Test Drug | F(5,60) = 7.70 | **p<0.0001** |
|  | Main Effect of Sex | F(1,60) = 0.53 | p=0.4690 |
|  | Drug x Sex Interaction | F(5,60) = 1.98 | p=0.0951 |
| Movement Counts | Main Effect of Test Drug | F(5,60) = 19.77 | **p<0.0001** |
|  | Main Effect of Sex | F(1,60) = 2.72 | p=0.1046 |
|  | Drug x Sex Interaction | F(5,60) = 0.52 | p=0.7607 |

**Supplemental Table 18.** Two-way Dose x Sex ANOVA results for effects of fentanyl on inhibition of gastrointestinal transit and respiratory depression (Figure 9, left panel). Significant p values are shown in bold in the right column. There was a significant Dose x Sex interaction for inhibition of gastrointestinal transit, and post hoc analysis indicated significantly greater inhibition in males at a dose of 0.032 mg/kg fentanyl (p=0.0020). There was also a significant Dose x Sex interaction for respiratory depression. Post hoc analysis did not identify a significant difference between sexes at any dose, but there was a trend for respiration to be depressed more in males than in females at 0.32 mg/kg fentanyl (p=0.0591).

| **Endpoint** | **Factor** | **F value** | **P value** |
| --- | --- | --- | --- |
| Gastrointestinal Inhibition | Main Effect of Dose | F(4,50) = 92.31 | **p<0.0001** |
|  | Main Effect of Sex | F(1,50) = 0.18 | p=0.6760 |
|  | Dose x Sex Interaction | F(4,50) = 4.28 | **p=0.0047** |
| Respiratory Depression | Main Effect of Dose | F(5,60) = 37.81 | **p<0.0001** |
|  | Main Effect of Sex | F(1,60) = 0.37 | p=0.5468 |
|  | Dose x Sex Interaction | F(5,60) = 2.605 | **p=0.0338** |

**Supplemental Table 19.** Two-way Proportion x Sex ANOVA results for effects of fentanyl/naltrexone proportions on inhibition of gastrointestinal transit and respiratory depression (Figure 9, center and right panel). Significant p values are shown in bold in the right column. There was not a significant main effect of Sex or a Proportion x Sex interaction on either endpoint.

| **Endpoint** | **Factor** | **F value** | **P value** |
| --- | --- | --- | --- |
| Gastrointestinal Inhibition | Main Effect of Proportion | F(6,70) = 53.90 | **p<0.0001** |
|  | Main Effect of Sex | F(1,70) = 0.07 | p=0.7882 |
|  | Proportion x Sex Interaction | F(6,70) = 1.95 | p=0.0853 |
| Respiratory Depression | Main Effect of Proportion | F(6,70) = 23.58 | **p<0.0001** |
|  | Main Effect of Sex | F(1,70) = 2.68 | p=0.1058 |
|  | Proportion x Sex Interaction | F(6,70) = 1.01 | p=0.4255 |
